# Radiation-induced lung damage promotes breast cancer lung-metastasis through CXCR4 signaling

**DOI:** 10.18632/oncotarget.5666

**Published:** 2015-09-15

**Authors:** Lynn Feys, Benedicte Descamps, Christian Vanhove, Anne Vral, Liv Veldeman, Stefan Vermeulen, Carlos De Wagter, Marc Bracke, Olivier De Wever

**Affiliations:** ^1^ Department of Radiation Oncology and Experimental Cancer Research, Laboratory of Experimental Cancer Research, Ghent University, Ghent, Belgium; ^2^ Department of Electronics and Information System, iMinds-IBiTech-MEDISIP, Ghent University, Ghent, Belgium; ^3^ Department of Basic Medical Sciences, Physiology Group, Ghent University, Ghent, Belgium; ^4^ Department of Radiation Oncology and Experimental Cancer Research, Gent University Hospital, Ghent, Belgium; ^5^ Department of Biomedical Science, HoGent, Ghent, Belgium

**Keywords:** radiotherapy, triple-negative breast cancer, CXCL12, MIF, AMD3100

## Abstract

Radiotherapy is a mainstay in the postoperative treatment of breast cancer as it reduces the risks of local recurrence and mortality after both conservative surgery and mastectomy. Despite recent efforts to decrease irradiation volumes through accelerated partial irradiation techniques, late cardiac and pulmonary toxicity still occurs after breast irradiation. The importance of this pulmonary injury towards lung metastasis is unclear. Preirradiation of lung epithelial cells induces DNA damage, p53 activation and a secretome enriched in the chemokines SDF-1/CXCL12 and MIF. Irradiated lung epithelial cells stimulate adhesion, spreading, growth, and (transendothelial) migration of human MDA-MB-231 and murine 4T1 breast cancer cells. These metastasis-associated cellular activities were largely mimicked by recombinant CXCL12 and MIF. Moreover, an allosteric inhibitor of the CXCR4 receptor prevented the metastasis-associated cellular activities stimulated by the secretome of irradiated lung epithelial cells. Furthermore, partial (10%) irradiation of the right lung significantly stimulated breast cancer lung-specific metastasis in the syngeneic, orthotopic 4T1 breast cancer model.

Our results warrant further investigation of the potential pro-metastatic effects of radiation and indicate the need to develop efficient drugs that will be successful in combination with radiotherapy to prevent therapy-induced spread of cancer cells.

## INTRODUCTION

Postoperative radiotherapy reduces the risk of both recurrence and mortality of breast cancer, and is nowadays standard treatment in the management of breast cancer after conservative surgery and after mastectomy to anticipate the high risk of relapse [1, 2]. Despite this progress, locoregional post radiotherapy relapses still occur in about 7-12.6% of the patients within the 5 years after treatment [3-5]. Relapses occurring within a preirradiated area are associated with an increased risk of local invasion, metastasis formation and poor prognosis compared to relapses occurring outside of the irradiated area [6]. Recent experimental evidence supports these clinical observations. In murine xenograft models, tumors developing within preirradiated beds are more invasive and metastatic compared to tumors growing outside irradiated beds, a condition also referred to as “tumor bed effect”. Kuonen et al. investigated cellular and molecular mechanisms underlying the tumor bed effect in breast cancer by using the 4T1 triple-negative murine model mimicking local relapse after radiotherapy and identified the role of cancer cells and mobilized myeloid cells as a metastasis promoting mechanism in breast [7]. Also, radiation-induced stemness of residual breast cancer cells increased spontaneous lung metastasis [8].

Although these experimental models adequately address the impact of the local tumor bed effect, these *in vivo* models do not consider the incidental exposure of the cardiopulmonary region to ionizing radiation after postoperative radiotherapy. Incidental cardiopulmonary irradiation is clinically important since it increases the subsequent rate of ischemic heart disease and secondary lung cancer risk [9, 10]. Radiotherapy regimens for breast cancer have changed since these trials; the doses of up to 15 Gy to which the cardiopulmonary region was exposed are now generally lower [9, 10]. Nevertheless, in most women receiving contemporary radiotherapy protocols, the cardiopulmonary region receives doses of 1 to 10.9 Gy [11]. The estimated percentage of total irradiated lung volume may range from 2.7 to 17.6% in a study population receiving tangential radiation beams [12].

Lungs are a prime target organ for breast cancer metastasis but the impact of incidental radiation exposure on lung metastasis is unknown. In this paper, we experimentally and molecularly addressed whether preirradiation of lung epithelial cells impacts metastasis-associated cellular activities of well-characterized triple-negative human MDA-MB-231 and murine 4T1 breast cancer cells. Using a murine xenograft model, lung metastasis formation was evaluated after exposure of 10% volume of the right lung to clinically relevant doses of radiation.

## RESULTS

### Radiation effects on damage response and senescence markers in normal lung microenvironments

**Figure 1:**
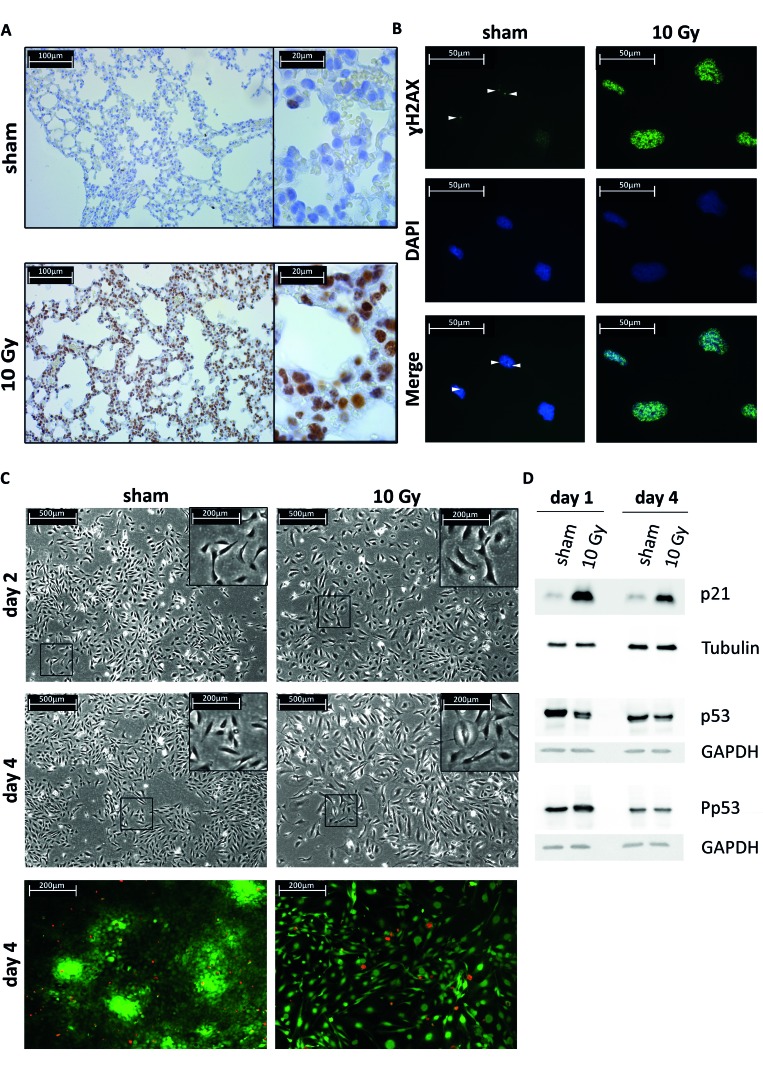
Lung epithelial cells radiation response and senescence markers **A.** Immunohistochemical (IHC) staining of γH2AX foci using an immunoenzymatic DAB staining method (brown color) combined with a haematoxylin counterstaining in sham or 10 Gy irradiated mouse lung tissue. **B.** Immunocytochemical (ICC) staining of γH2AX foci (Alexa488 labeled secondary antibody, green color) combined with a DAPI nuclear counterstaining (blue color) in sham or 10 Gy irradiated Beas-2B lung epithelial cells. **C.** Upper 4 panels, phase contrast micrographs of Beas-2B lung epithelial cells two or four days post sham or 10 Gy irradiation. The 10 Gy condition shows less dense cell culture, a more spread cell morphology with enlarged nuclei and increased cytoplasmic surface area. Lower 2 panels, live/dead - viability/cytotoxicity test. Assay shows live cells as green and dead cells as red. Four days after single irradiation dose of 10 Gy shows no increase of Beas-2B cell death. **D.** Western blot (WB) analysis of p53 and p21 on total cell lysates from Beas-2B cells treated with single-fraction 10 Gy or sham. Total p53 expression is unchanged after irradiation but increase in p53 phosphorylation is observed at day 1 after treatment and normalizes at day 4. Total expression of p21 is increased until day 4. GAPDH and tubulin are used as loading control.

To assess treatment-induced damage response in normal cells of the lung microenvironment, we examined mouse lung tissue that was excised 15 minutes after receiving thoracic sham or 10 Gy irradiation. We found evidence of DNA damage in lung epithelial cells as determined by the phosphorylation of histone H2AX on Ser139 (γH2AX) within 15 minutes after 10 Gy irradiation (Figure 1A). To further ascertain the consequence of DNA damage in benign cells, we established an *in vitro* model treating Beas-2B epithelial cells of the lung microenvironment with a 10 Gy single radiation dose which substantially increased the number of γH2AX foci (Figure 1B). Irradiated cells showed no increase in cell death (Figure 1C, lower panel), but showed a more spread morphology with enlarged nuclei and increased cytoplasmic surface area (Figure 1C, upper panel). Furthermore, activation of p53 and increased expression of the p21 cell cycle arrest protein were observed (Figure 1D, Supplementary Figure S1). An indicator of cellular senescence, p21, was maintained up to 4 days after irradiation, which explains the lower number of cells (Figure 1B, 1C and 1D).

### Impact of irradiated lung epithelial cells on breast cancer cell growth and adhesion

**Figure 2:**
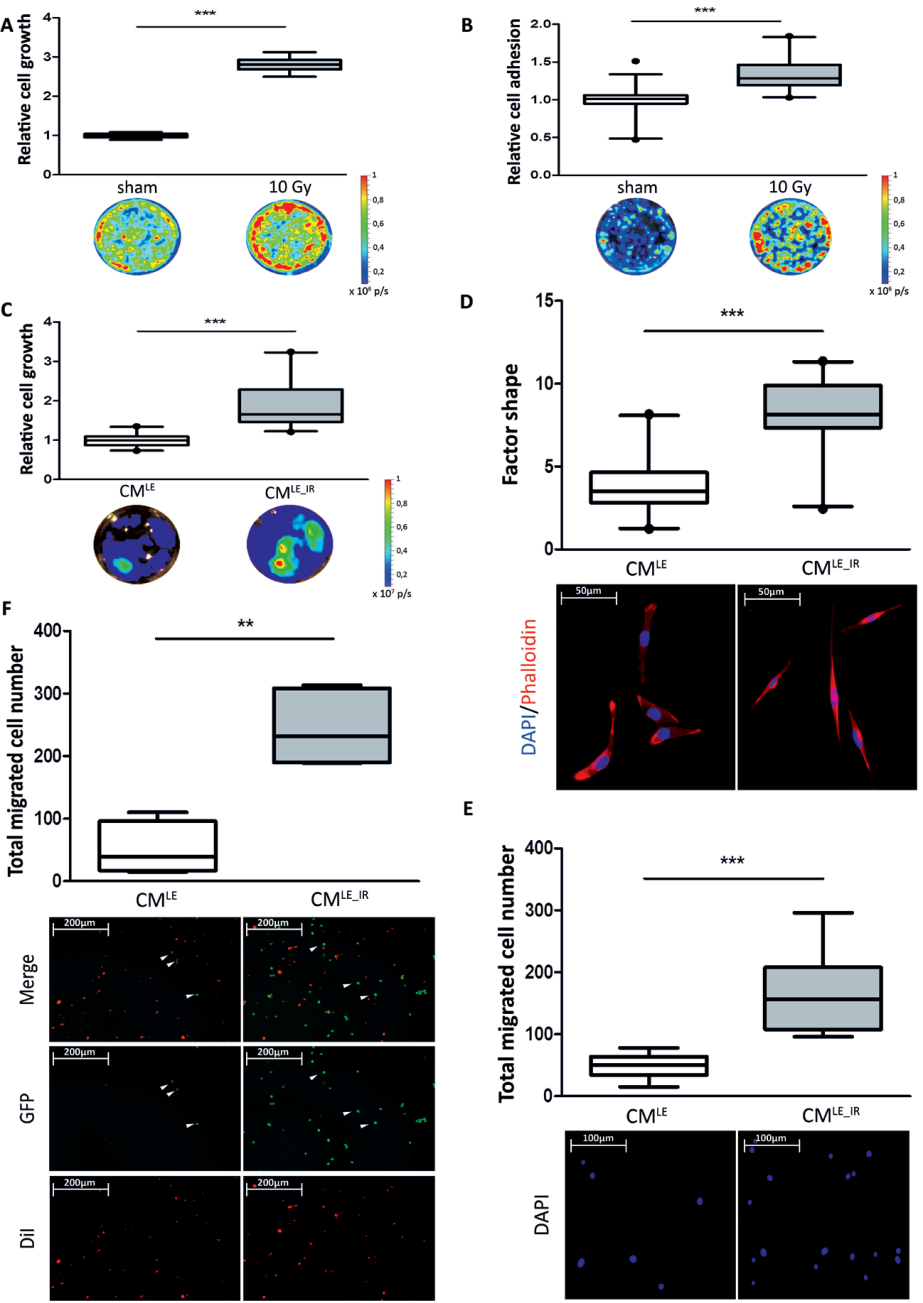
Impact of irradiated lung epithelial cells on breast cancer cell adhesion and growth **A.** Box plots illustrating the relative cell growth of MDA-MB-231GFP_luc cells. Co-culture of breast cancer with irradiated Beas-2B cells increases relative cell growth compared to co-culture with sham treated Beas-2B cells. Quantification by bioluminescent imaging after 4 days incubation. Data is represented as relative fold change compared with the corresponding control value. MDA-MB-231GFP_luc: *n* = 6; ***, *P* < 0.001 (Unpaired *t*-test with Welch's correction). **B.** Box plots illustrating the relative cell adhesion of MDA-MB-231GFP_luc cells. Relative Adhesion of breast cancer cells to irradiated Beas-2B cell monolayer is increased compared to sham treated Beas-2B cell monolayer. Quantification by bioluminescent imaging after 24 hours incubation. Data is represented as relative fold change compared with the corresponding control value. MDA-MB-231GFP_luc: *n* = 15; ***, *P* < 0.0001; circle = outlier (Unpaired *t*-test with Welch's correction). *Impact of CM^LE_IR^ on breast cancer cell morphology, growth, migration and extravasation*. **C.** Box plots illustrating the relative cell growth of MDA-MB-231GFP_luc cells. Treatment of breast cancer cells with CM^LE_IR^ increases relative cell growth compared to cells treated with CM^LE^. Quantification by bioluminescent imaging after 4 days incubation. Data is represented as relative fold change compared with the corresponding control value (CM^LE^). MDA-MB-231GFP_luc: *n* = 21; ***, *P* < 0.001 (Mann-Whitney U). **D.** Box plots illustrating the extent of cell spreading of MDA-MB-231GFP_luc cells in CM^LE_IR^ versus CM^LE^ conditions, as quantified by factor shape (upper panel). Treatment with CM^LE_IR^ showed enhanced cell spreading, corresponding with the significantly altered cell shapes (lower panel). Fluorescence microscopy images of cells double stained with phalloidin for actin filaments (red) and DAPI counterstaining for nuclei (blue) after 4 days of incubation with CM^LE_IR^ versus CM^LE^. *n* = 20; ***, *P* < 0.001 (Mann-Whitney U). **E.** Box plots illustrating total migrated cell number of MDA-MB-231GFP_luc cells in CM^LE_IR^ versus CM^LE^ conditions (upper panel). Nuclei of migrated cells were stained blue by DAPI (lower panel). *n* = 12; ***, *P* < 0.001 (Unpaired *t*-test with Welch's correction). **F.** Box plots illustrating total migrated cell number of MDA-MB-231GFP_luc cells through endothelial monolayer in CM^LE_IR^ versus CM^LE^ conditions (upper panel). CM^LE_IR^ enhances breast cancer cell extravasation significantly. Endothelial cells were stained red with Vibrant DiI. Extravasated MDA-MB-231GFP_luc cells are green (lower panel). *n* = 6; **, *P* = 0.0022 (Mann-Whitney U). Arrowheads indicating GFP positive migrated breast cancer cells.

Irradiated or sham-irradiated Beas-2B cells were grown in co-culture with 4T1_luc or MDA-MB-231GFP_luc triple-negative breast cancer cells and cancer cell growth was monitored by measuring luciferase activities after 4 days of co-culture. Co-culture with irradiated Beas-2B cells significantly enhanced the relative cancer cell growth 1.7-and 2.8-fold respectively compared to co-culture with unirradiated Beas-2B cells (4T1_luc: sham *vs*. 10 Gy: 1.000 ± 0.030 *vs*. 1.740 ± 0.172; *P* < 0.001; MDA-MB-231GFP_luc: sham *vs*. 10 Gy: 1.000 ± 0.067 *vs*. 2.806 ± 0.203; *P* < 0.001) (Supplementary Figure S2A, Figure 2A).

To study the effect on cancer cell adhesion, we seeded breast cancer cells on a monolayer of Beas-2B epithelial cells 24 hours after irradiation or sham-irradiation. Co-culture with irradiated Beas-2B cell monolayer significantly increased adhesion of both cancer cells 1.7-and 1.3-fold respectively compared to co-culture with unirradiated Beas-2B cell monolayer (4T1_luc: sham *vs*. 10 Gy: 1.000 ± 0.068 *vs*. 1.66 ± 0.3211; *P* < 0.001; MDA-MB-231GFP_luc: sham *vs*. 10 Gy: 1.000 ± 0.182 *vs*. 1.328 ± 0.210; *P* < 0.001) (Supplementary Figure S2B, Figure 2B).

### Impact of soluble factors derived from irradiated lung epithelial cells on breast cancer cell morphology, growth, migration and extravasation

To further investigate these effects we collected conditioned medium of unirradiated (CM^LE^) and irradiated Beas-2B cells (CM^LE_IR^). Incubation of 4T1_luc and MDA-MB-231GFP_luc cells with CM^LE_IR^ significantly increased relative cell growth 1.8-and 1.9-fold respectively compared to incubation with CM^LE^ (Supplementary Figure S2C, Figure 2C) (CM^LE^*vs*. CM^LE_IR^; 4T1_luc: 1.000 ± 0.314 *vs*. 1.800 ± 0.730; *P* < 0.001; MDA-MB-231GFP_luc: 1.001 ± 0.159 *vs*. 1.891 ± 0.569; *P* < 0.001).

F-Actin staining of single cells revealed a more elongated morphology upon CM^LE_IR^ compared to CM^LE^ (Figure 2D, lower panel). This is shown by an increase in mean factor shape of MDA-MB-231GFP_luc cells incubated with CM^LE_IR^, this was 2.2-fold higher compared to the CM^LE^ condition (CM^LE^*vs*. CM^LE_IR^: 3.742 ± 1.686 *vs*. 8.240 ± 2.197; *P* < 0.001) (Figure 2D, upper panel).

Morphological changes suggest an impact on the migratory potential of cells. To investigate the effect of irradiated epithelial cells on breast cancer cell migration we employed TransWell® culture chambers separated into two compartments by microporous filters. In the lower compartment CM^LE^ or CM^LE_IR^ was added, while on top MDA_MB-231GFP_luc cells were seeded. As shown in Figure 2E, the directed migration of MDA-MB-231GFP_luc cells was significantly increased by 3.4-fold in the cells in presence of CM^LE_IR^ compared to those of CM^LE^ (CM^LE^*vs*. CM^LE_IR^: 49.46 ± 18.52 cells *vs*. 166.70 ± 60.75 cells; *P* < 0.001).

Extravasation, the migration of cancer cells through the endothelial wall into the target parenchyma, is another critical step in metastasis. This functional activity was biomimicked by studying directed breast cancer cell migration to CM^LE^ or CM^LE_IR^ through a monolayer of endothelial cells. Under CM^LE_IR^ conditions 4.7-fold more MDA-MB-231GFP_luc migrated through the endothelial layer compared to CM^LE^ conditions (CM^LE^*vs*. CM^LE_IR^: 52.00 ± 39.69 cells *vs*. 243.70 ± 57.25 cells; *P* = 0.002) (Figure 2F, Arrowheads in CM^LE^ point to migrated cancer cells.).

Summarized, we have shown that the secretome of irradiated Beas-2B lung epithelial cells contains factors that reorganize breast cancer cells to a more elongated shape and increase growth, migration and extravasation of the cancer cells.

### Increased secretion of CXCL12 and MIF by irradiated lung epithelial cells

**Figure 3:**
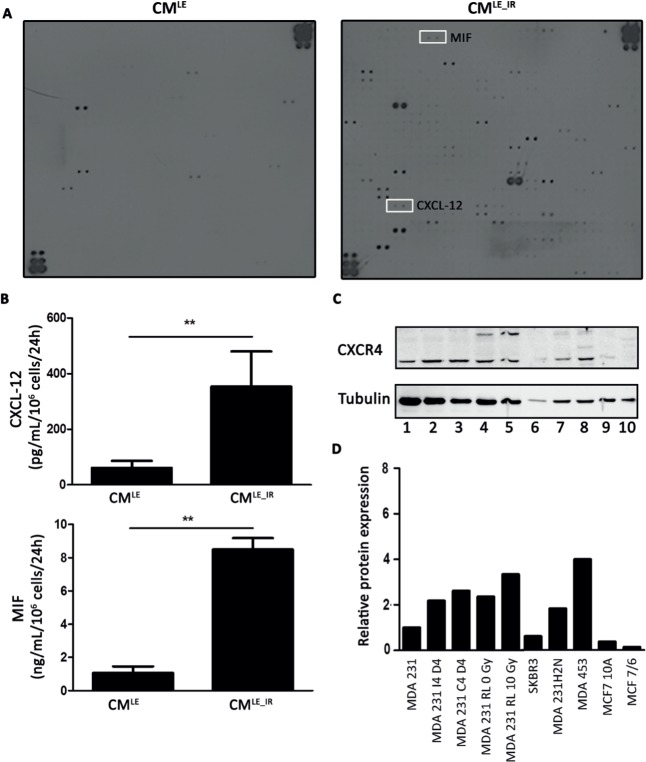
Increased secretion of CXCL12 and MIF by irradiated lung epithelial cells **A.** Cytokine array identifies enhanced presence of CXCL12 and MIF in CM^LE_IR^ compared to CM^LE^. **B.** ELISA analysis of CXCL12 (upper panel) and MIF (lower panel) on CM^LE_IR^ versus CM^LE^. For each condition 2 different samples were used in triplicate. Values are presented as the mean ± SD. CXCL12: **, *P* = 0.010. MIF: **, *P* = 0.004 (Mann-Whitney U). **C.** Western blot analysis of CXCR4 expression on total lysate of breast cancer cells. Lane 1-5, represents MDA-MB-231GFP_luc (invasive cell line) in different conditions (MDA 231: parental MDA-MB-231GFP_luc; MDA 231 I4 D4: MDA 231 cells exposed to CM^LE_IR^ for 4 days; MDA 231 C4 D4: MDA 231 cells exposed to CM^LE^ for 4 days; MDA 231 RL 0 Gy: MDA 231 isolated from non-irradiated mouse lung; MDA 231 RL 10 Gy: MDA 231 isolated from irradiated mouse lung. Lane 6: SKBR3 (non-invasive cell line). Lane 7: MDA-MB-231H2N (invasive cell line). Lane 8: MDA-MB-453 (invasive cell line). Lane 9: MCF 10A (non-invasive cell line). Lane 10: MCF7/6 (non-invasive cell line). **D.** Quantitative analysis CXCR4 protein expression level. Western blot analysis of CXCR4 protein expression in different breast cancer cell lines after tubulin normalization. Protein levels relative to control, MDA-MB-231GFP_luc.

The composition of CM^LE^ and CM^LE_IR^ was assessed to determine which cytokines were secreted by unirradiated versus irradiated lung epithelial cell. Semi-quantitative results from a cytokine array showed that CM^LE_IR^ contained a total of 52 cytokines with a signal that exceeded that of CM^LE^ condition (Figure 3A; Supplementary Table S1). We selected the CXCL12 and MIF cytokines for further study because of a high fold change (more than 80-fold) and a known role in breast cancer metastasis [13]. CXCL12 and MIF, which had an 83.88-and 86.46-fold higher presence, respectively, in CM^LE_IR^ compared to CM^LE^ (Supplementary Table 1). Responding to fold changes (relative changes) rather than absolute change is intrinsically important in chemokine attraction and consequently regulation of metastasis [14, 15]. Quantitative ELISA data showed that irradiated lung epithelial cells had a secretion of CXCL12 and MIF that is 5.8-and 7.9-fold higher, respectively, than unirradiated lung epithelial cells (Figure 3B) (CM^LE^*vs*. CM^LE_IR^; CXCL12: 61.07 ± 12.59 *vs*. 353.70 ± 126.00 pg/mL/10^6^ cells/24h; *P* = 0.010; MIF: 1.072 ± 0.390 *vs*. 8.495 ± 0.695 ng/mL/10^6^ cells/24h; *P* = 0.004). According to previous literature, both cytokines may affect metastasis through activation of the CXCR4 receptor on cancer cells [13]. Western Blot analysis confirmed that cancer cell lines, that are known to have an invasive phenotype, have a higher expression of CXCR4 (45-47 kDa) (Figure 3C-3D) [16].

### Effect of recombinant CXCL12 and MIF on breast cancer cell growth and migration

**Figure 4:**
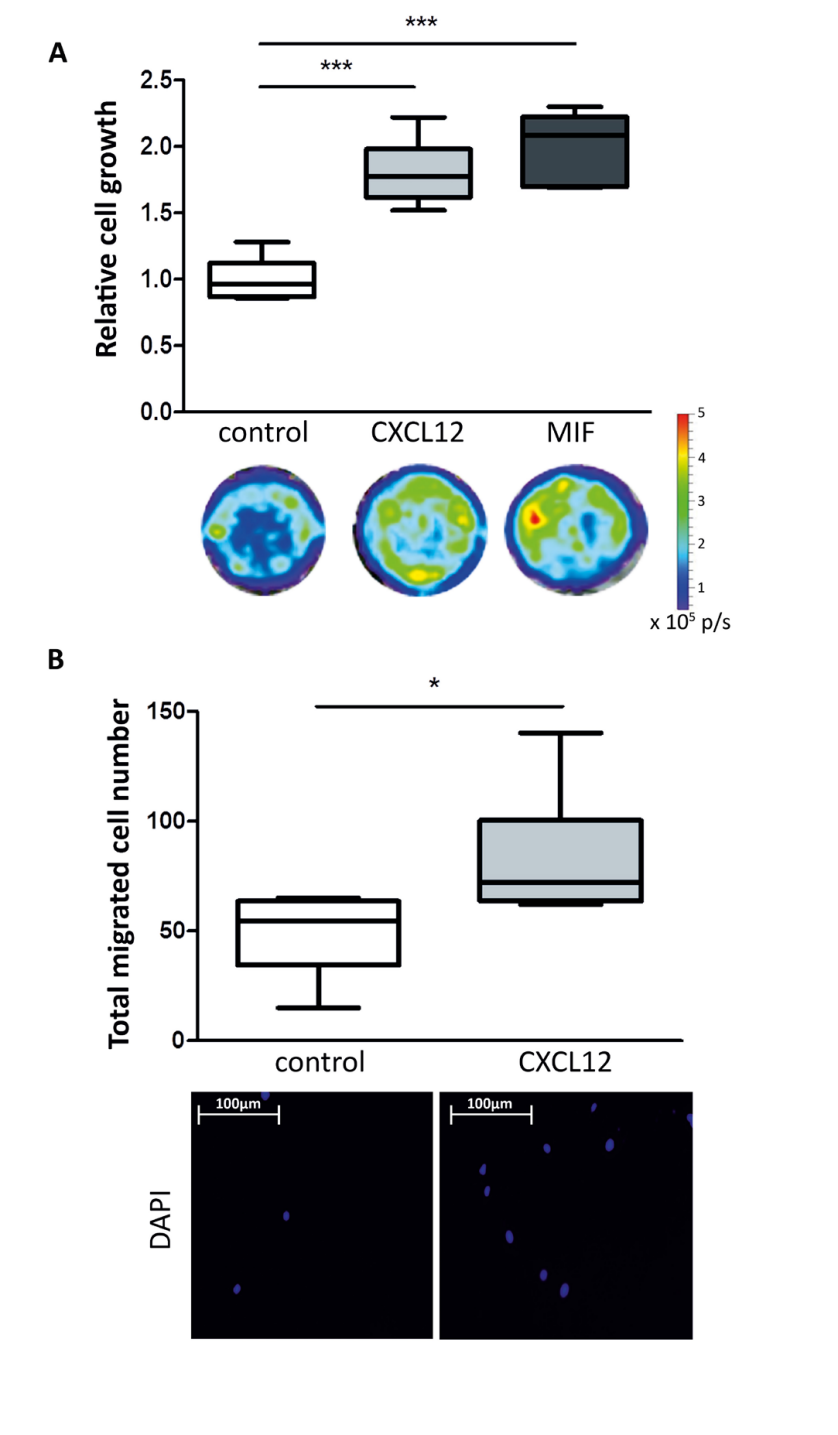
Effect of recombinant CXCL12 and MIF on breast cancer cell growth and migration **A.** Box plots illustrating the relative cell growth of MDA-MB-231GFP_luc cells. Treatment of breast cancer cells with CXCL12 (50 ng/mL) or MIF (50 ng/mL) increases relative cell growth compared to control. Quantification by bioluminescent imaging after 4 days incubation. Data is represented as relative fold change compared with the corresponding control value (CM^LE^). *n* = 6; ***, *P* < 0.001 (Unpaired *t*-test). **B.** Box plots illustrating difference in total migrated cell number of MDA-MB-231GFP_luc cells treated with CXCL12 (50 ng/mL) (upper panel). Nuclei of migrated cells were stained blue with DAPI (lower panel). *n* = 6; *, *P* = 0.038 (Unpaired *t*-test).

To verify if CXCL12 and MIF may contribute to the effects observed with the secretome of irradiated cells, the recombinant forms were supplemented to the secretome of unirradiated cells and used to assess the functional impact on cancer cells. CM^LE^ supplemented with recombinant CXCL12 or MIF induced an increase in cell growth as demonstrated for MDA-MB-231GFP_luc cells (Figure 4A) (CM^LE^*vs*. CM^LE^ + CXCL12: 1.002 ± 0.157 *vs*. 1.807 ± 0.241; *P* < 0.001; CM^LE^*vs*. CM^LE^ + MIF: 1.002 ± 0.157 *vs*. 2.010 ± 0.259; *P* < 0.001) and induced an increase in cell migration after treatment with CXCL12 (Figure 4B) (CM^LE^*vs*. CM^LE^ + CXCL12: 48.830 ± 18.640 *vs*. 82.830 ± 29.360; *P* = 0.038). Experiments with MIF did not show significant difference in cancer cell migration (data not shown).

### Allosteric targeting of CXCR4 receptor reversed paracrine effect induced by irradiated epithelial cells

**Figure 5:**
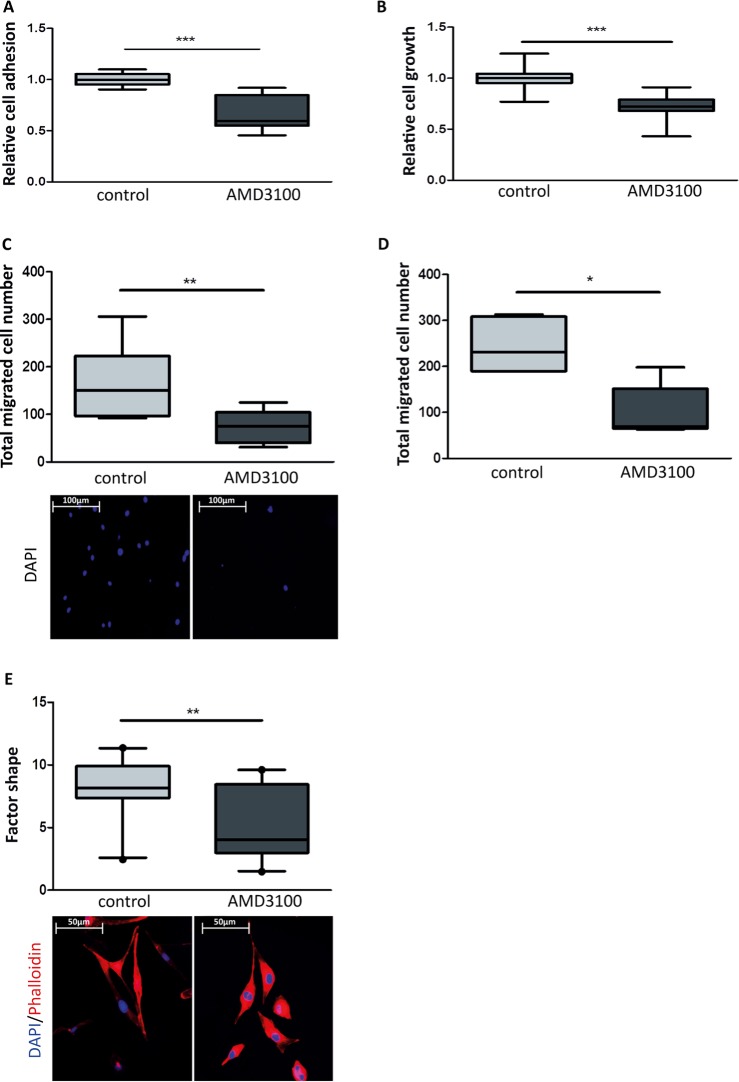
Effect of an allosteric CXCR4 inhibitor on breast cancer cell growth and migration **A.** Box plots illustrating the relative cell adhesion of MDA-MB-231GFP_luc cells. Treatment of breast cancer cells with AMD3100 (10 μM) decreases relative cell adhesion to irradiated Beas-2B cell monolayer. *n* = 12; ***, *P* < 0.001(Unpaired *t*-test with Welch's correction). **B.** Box plots illustrating the relative cell growth of MDA-MB-231GFP_luc cells. Treatment of breast cancer cells with AMD3100 (10 μM) decreases relative cell growth in presence of CM^LE-IR^. (Unpaired *t*-test with Welch's correction). *n* = 15; ***, *P* < 0.001 (Unpaired *t*-test). In **C.** and **D.**, quantification by bioluminescent imaging after 4 days incubation. Data is represented as relative fold change compared with the corresponding control value. **C**. Box plots illustrating impact of AMD3100 (10 μM) on total migrated cell number of MDA-MB-231GFP_luc cells stimulated by CM^LE-IR^. Nuclei were stained blue with DAPI (lower panel). *n* = 9; **, *P* = 0.009 (Unpaired *t*-test with Welch's correction). **D**. Box plots illustrating total transendothelial migrated cell number of MDA-MB-231GFP_luc cells stimulated by CM^LE-IR^ in presence of AMD3100 (10 μM) or control. *n* = 6; *, *P* = 0.017 (Mann-Whitney U). **E.** Box plots illustrating the extent of CM^LE-IR^ -induced cell spreading of MDA-MB-231GFP_luc cells treated with AMD3100 (10 μM) versus control, as quantified by factor shape (upper panel). Fluorescence microscopy images of cells double stained with phalloidin for actin filaments (red) and DAPI counterstaining for nuclei (blue) (lower panel). *n* = 20; **, *P* = 0.004 (Mann-Whitney U).

To investigate whether the observed effects depend on activation of the CXCR4 receptor, AMD3100, an allosteric inhibitor of CXCR4 receptor, was used. Addition of AMD3100 reversed the pro-metastasis associated effects of CM^LE_IR^ such as relative cell growth (Figure 5A) (CM^LE_IR^*vs*. CM^LE_IR^ + AMD3100: 1.000 ± 0.107 *vs*. 0.717 ± 0.144; *P* < 0.001 ), relative adhesion (Figure 5B) (CM^LE_IR^*vs*. CM^LE_IR^ + AMD3100: 1.000 ± 0.060 *vs*. 0.665 ± 0.166; *P* < 0.001), migration and extravasation of breast cancer cells (Figure 5C & 5D) (Migration - CM^LE_IR^*vs*. CM^LE_IR^ + AMD3100: 162.40 ± 74.62 cells *vs*. 75.33 ± 33.32 cells; *P* = 0.009;Extravasation - CM^LE_IR^*vs*. CM^LE_IR^+ AMD3100: 243.70 ± 57.25 cells *vs*. 100.40 ± 57.11 cells; *P* = 0.017). Furthermore, AMD3100 treatment partially reversed CM^LE_IR^ -induced morphological changes, as measured by factor shape (Figure 5E) (CM^LE_IR^*vs*. CM^LE_IR^ + AMD3100: 8.240 ± 2.197 *vs*. 5.243 ± 2.772; *P* = 0.004).

### Paracrine activation of ERK, Akt and STAT3 in breast cancer cells by CM^LE_IR^ and recombinant CXCL12

**Figure 6:**
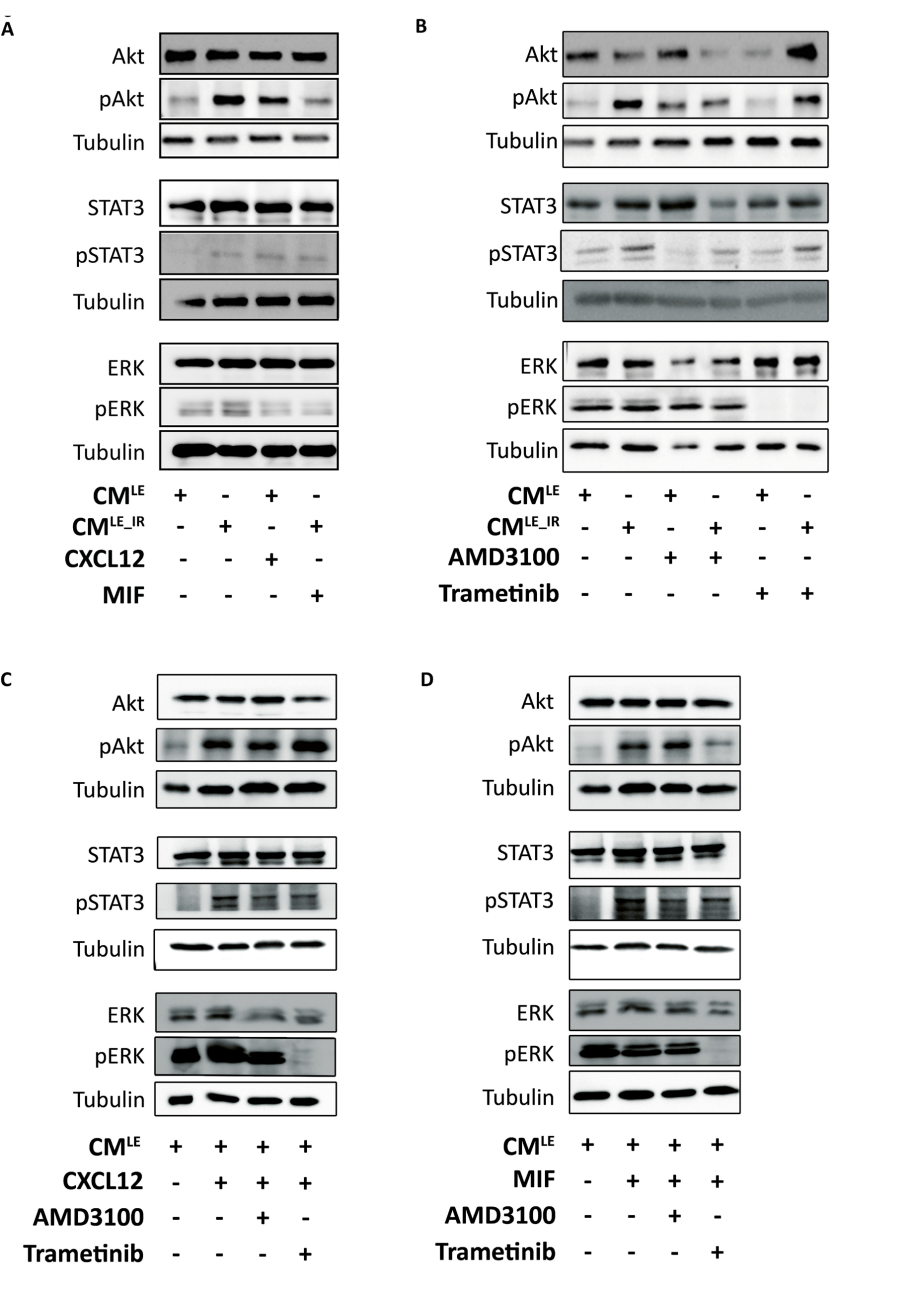
Paracrine activation of ERK, Akt and STAT3 in breast cancer cells by CM^LE_IR^ and recombinant CXCL12 **A.**-**D.**. Western blot analysis of pERK, pAkt and pSTAT3 in MDA-MB-231GFP_luc cells treated with recombinant CXCL12 (50 ng/mL) or MIF (50 ng/mL) **A.** CM^LE^, CM^LE_IR^, in presence of AMD3100 (10 μM) or Trametinib (50 nM) **B.** recombinant CXCL12 (50 ng/mL) **C.** or MIF (50 ng/mL) **D.** in presence of AMD3100 (10 μM) or Trametinib (50 nM) Tubulin is used as loading control.

CM^LE_IR^ activated multiple CXCR4-dependent downstream cascades, like ERK, Akt and STAT3, in MDA-MB-231GFP_luc cells (Figure 6A). These pathways are involved in mediating cellular proliferation, survival, migration, transformation and differentiation [17]. In agreement, addition of recombinant CXCL12 or MIF stimulated Akt, ERK and STAT3 activation, although an equal concentration of MIF showed a smaller increase than CXCL12 (Figure 6A, Supplementary Figure S3A).

Next, we examined the impact of Trametinib and AMD3100 on CM^LE_IR^-induced ERK, Akt and STAT3 activation. Previous literature showed that triple-negative breast cancer cells are the most sensitive for treatment with MEK inhibitor[18]. Nanomolar concentrations of Trametinib, an allosteric MEK1/2 inhibitor, completely blocked CM^LE_IR^ induced ERK activation (Figure 6B, Supplementary Figure S3B) with no impact on AKT or STAT3 activation. Similar effects were observed when combining recombinant CXCL12 or MIF with Trametinib (Figure 6C-6D, Supplementary Figure S3C-D). In accordance with the functional experiments, AMD3100 decreased CM^LE_IR^-induced Akt and STAT3 pathway activation; a minor impact on pERK was observed in the cancer cells exposed with CM^LE_IR^ + AMD3100 compared to CM^LE_IR^ alone (Figure 6B). These effects with AMD3100 are less pronounced when CM^LE^ is combined with recombinant CXCL12 and MIF (Figure 6C-6D, Supplementary Figure S3C-D).

### Impact of partial lung irradiation on lung-specific breast cancer metastasis in a syngeneic mouse model

**Figure 7:**
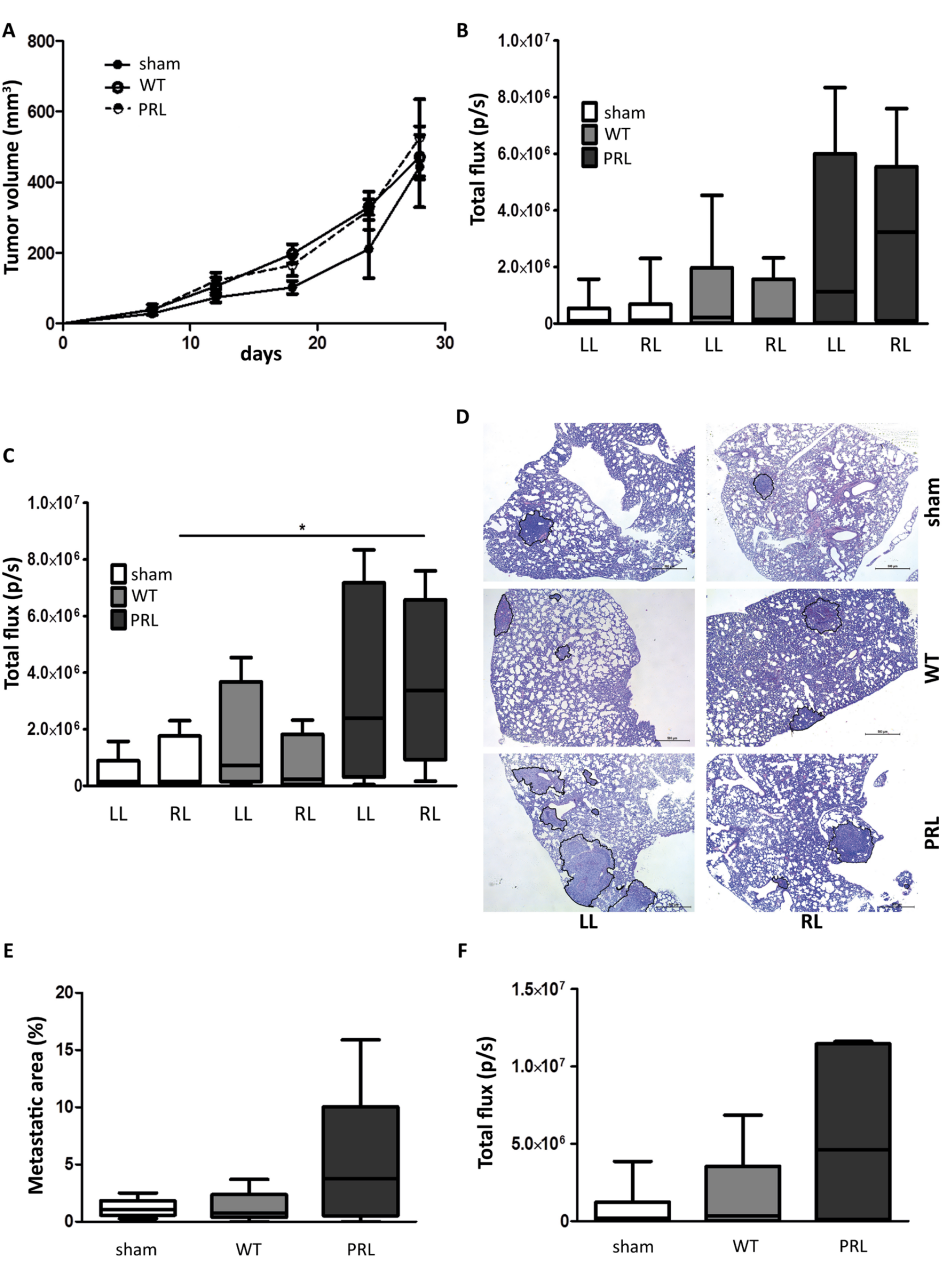
Impact of partial lung irradiation on lung-specific breast cancer metastasis in a syngeneic mouse model **A.** 4 week old BALB/c female mice were injected orthotopically with 1 × 10^6^ 4T1_luc cells in 0.1 mL of serum free DMEM with 50% Matrigel. Tumor formation was monitored for 28 days by caliper measurement. Tumor volumes were measured as indicated. Sham and WT IR: *n* = 6 and PRL: *n* = 5. **B.**-**C.** & **F.** 28 days after implantation of the cells, mice were sacrificed and total lung metastasis was quantified by bioluminescent imaging. Lung were quantified separately (B & C) or as one (F). Sham and WT: LL and RL, *n* = 6 and PRL: LL and RL, *n* = 5 (B). Only lungs containing metastasis were quantified, showing significant increase in signal in right lung of PRL mice. Sham: LL, *n* = 5; RL, *n* = 4; WT: LL, *n* = 4; RL, *n* = 5; PRL: LL, *n* = 4; RL, *n* = 4; *; *P* = 0.043 (Man-Whitney U test) (C). **D.** H&E staining of lungs from indicated groups. The metastatic areas are encircled (black). **E.** Quantification of the percentage of lung metastatic area calculated per mouse. Sham and WT: *n* = 6 and PRL: *n* = 5.

A syngeneic, orthotopic triple-negative breast cancer model 4T1_luc was developed to study the impact of irradiation of the lung on the formation of lung metastasis. Patient studies showed that approximately 10% of the total lung volume is irradiated during breast cancer RT, with a mean lung dose of 10 Gy [11, 12]. All groups of mice received CT scan radiation for the localization of lung tissue; in the sham treatment group, no further irradiation was performed; the WT group received a 10 Gy irradiation to the whole thorax; the PRL group (partial right lung) received a 10 Gy irradiation to approximately 18mm^3^ part (10% of total volume) of the right lung (Supplementary Figure S4-S5). 4T1_luc cells (1 × 10^6^) were orthotopically injected 24 hours after irradiation. Neither WT nor PRL irradiation did significantly impact primary tumor growth (Figure 7A) (sham *vs*. 100% IR *vs*. 10% IR: 446 ± 111 mm^3^ *vs*. 471 ± 63 mm^3^ *vs*. 526 ± 109 mm^3^). Four weeks after orthotopic 4T1_luc breast cancer inoculation, all lungs were prelevated and bioluminescent activity in the separate lungs was compared between the three groups. Mice which received a 10 Gy PRL irradiation showed more bioluminescent signal, and thus more metastasis, in each lung compared to WT irradiated or sham treated mice (Figure 7B). In addition, when only bioluminescent signal of those lungs with activity were included we observed significantly higher signals in the right lung of PRL irradiated mice compared to the right lung of sham treated mice, suggesting more metastatic growth (*P* = 0.043) (Figure 7C, Supplementary Figure S6); a similar trend was observed for the left lung but no significant value was reached. Semi-quantitative histological examination of metastatic lung tissue demonstrated more and larger metastasis in mice receiving PRL irradiation compared to sham or WT (Figure 7D, as illustration. Figure 7E, quantification). This was confirmed by quantitatively analyzing the total metastatic area between the groups. In sham groups an average metastatic area of 1.2% was reached, comparable to the WT irradiated group with an average area of 1.3%. Both groups are remarkably, but not significantly, lower than the PRL irradiated group, with a three-fold increase of metastatic area (5.0%) (Figure 7E). Moreover, comparing total lung bioluminescent signal (signal right and left lung as one), sham treated mice still had the lowest signal but WT irradiated mice and PRL irradiated mice showed a 2.1- and 7.1-fold increase, respectively, in signal (Figure 7F).

## DISCUSSION

Because of the absence of oestrogen-, progesterone- and HER2 receptor [19], triple-negative breast cancers are not curable with hormonal treatment and HER2 therapies. Standard mastectomy or breast conserving surgery with adjuvant radio-and chemotherapy is used as a standard to treat this patients [20]. Postoperative radiotherapy of breast cancer reduces the risk of local recurrence and mortality after both conservative surgery and mastectomy [1, 2]. Despite recent efforts to decrease irradiation volumes and improved irradiation techniques, late cardiac and pulmonary toxicity does still occur after breast irradiation [6, 11]. The implications of this pulmonary injury for lung metastasis are unclear. A randomized trial in high-risk post-mastectomy patients showed that the long-term probabilities of lung metastases are significantly lower in the irradiated (RT) patients compared to non-irradiated (no-RT) patients. Interestingly, distant metastasis as first failure (independent from local relapse) was 47% in the RT group compared to 37% in the no-RT group, suggesting that lung metastasis do not necessarily occur after local relapse [21]. Although it is generally assumed in this case that micro-metastasis in the lungs were present before breast cancer was treated, an alternative, admittedly provocative, hypothesis is that radiation-induced collateral damage of the lung influences lung-specific metastasis. Metastasis can be an early event and stay undetected at time of diagnosis [22, 23]. Patients, presenting with micro-metastasis, receive radiotherapy which can stimulate metastatic growth. The difference in our *in vivo* model is that we first irradiated the lung and waited for metastasis to grow [22]. Our model is not relevant for the effect of ionizing radiation on already established micro-metastasis because we graft syngeneic breast cancer cells after ionizing radiation. We radiate part of the right lung, graft syngeneic breast cancer cells and study the impact on spontaneous lung metastasis formation. Lung irradiation has no impact on orthotopic tumor growth. However, we observe more and bigger metastatic foci in the lungs of PRL mice which can be caused by increased attraction of circulating cancer cells and/or stimulation of cancer cell release from the primary tumor and/or preparation of the metastatic niche (promoting adhesion and colonization of breast cancer cells). Breast cancer patients who did receive post-mastectomy radiotherapy showed most often distant metastasis as first site of failure in contrast to patients who did not receive post-mastectomy radiotherapy, here locoregional relapse and distant metastasis as first site of failure were equally common [21]. Studies (using post-mortem samplings) should be designed to investigate a potential relationship between collateral lung irradiation-damage and lung metastasis in breast cancer patients. The literature provides no level-one evidence because no randomized trials have been done correlating the dose indices of irradiated lung volumes with lung-specific metastasis in breast cancer. A clinical study with breast cancer patients undergoing postoperative radiation therapy of 50 Gy at 2 Gy/fraction, 5 days/week showed that the pulmonary region received a mean lung dose of approximately 10 Gy. If more than 3 axillary lymph nodes were affected, a supraclavicular field was added with equal dose and fractionation resulting in a higher mean pulmonary dose of 15.8 Gy [11]. This collateral irradiation to the lungs resulted in reduced pulmonary function in the first two years after the postoperative radiotherapy.

Histone H2AX phosphorylation is a recognized marker of DNA damage, i.e. double strand breaks [24]. The manner how DNA responds to radiation damage, indicated by γH2AX foci, has been investigated in mice models receiving thoracic radiation and shown to be correlative with fibrosis in distressed mice [25]. In our experiments, sham-irradiated lung tissue sections of BALB/c mice were almost completely negative for γH2AX foci, whereas 10 Gy thoracic irradiation resulted in a massive increase in the number of pulmonary nuclei with γH2AX foci throughout the tissue sections, irrespective of cell type. *In vitro* experimentation on lung epithelial cells, the major pulmonary cell type, revealed potent H2AX and p53 responses to radiation which coincided with morphological changes, induction of cellular senescence markers and an enhanced secretion of multiple chemokines and growth factors. Gunjal et al. demonstrated that ovarian cancer cells responding to heat-sensitive chemoattractants released from irradiated organs, including the lung, are more migratory and metastatic [26]. Our *in vitro* irradiation experiment confirmed the increased release of CXCL12 and MIF upon irradiation. The heat-sensitive chemokines CXCL12 and MIF, both known to promote breast cancer metastasis, showed the highest fold change in secretion by irradiated lung epithelial cells compared to control, which was confirmed in our study [13, 27]. The irradiation caused induction of a metastasis-receptive microenvironment that promotes trafficking and homing of cancer cells to the lung [17, 27-30]. Once the cells arrive in the lung, increased levels of CXCL12 and MIF retain the breast cancer cells in lung and provides it with survival and growth factors, so metastatic growth is enhanced [31-35]. It is not known whether CXCL12 and MIF are increased in lungs of irradiated breast cancer patients but future research could investigate the sputum, serum or urine of breast cancer patients in the acute or long-term response to radiation. Müller and co-workers were the first to demonstrate CXCR4-mediated metastasis of breast cancer cells to CXCL12-rich environments, like bone marrow, brain, lungs and liver [13]. Elevated CXCR4 expression in breast cancer cells negatively correlates with overall survival and disease-free survival in breast cancer patients and is correlated with malignant breast stem cell activity [36, 37]. Targeting this pathway could thus be a promising therapeutic addition to radiotherapy. To investigate this, we used an allosteric inhibitor of CXCR4, AMD3100 or Plerixafor®. AMD3100 was originally developed for HIV treatment and is nowadays used in combination with G-CSF as a stem cell mobilizer in patients with multiple myeloma and lymphoma [9, 13, 38-40]. Our co-culture findings showed that treatment of breast cancer cells with AMD3100 blocks the functional and biochemical effects induced by the secretome of irradiated epithelial cells. Nevertheless, the toxic effects of AMD3100 in long-term treatment limits its clinical potential [41]. However, Peng et al. identified a small cyclic peptide (LY2510924) that inhibits CXCL12 and CXCR4 interaction and downstream signaling and function [42]. Currently, this peptide is included in clinical trials on patients with advanced cancer [43]. The CXCL12 analog peptide CTCE-9908 inhibits lung metastasis in mouse models [44, 45] and tests in Phase I/II clinical trials in cancer patients showed no major adverse effects [46]. Furthermore, the anti-CXCL12 aptamer NOX-A12 inhibits brain tumor recurrences after irradiation in rats [47]. Potentially favorable collateral inhibition of the CXCR4-CXCL12 axis may prevent lung fibrosis, improving the quality of life in breast cancer patients [48].

We are not the first to report radiotherapy-stimulated relapse in preclinical models. Ohuchida et al. reported that irradiation of stromal pancreatic fibroblast increased invasiveness of pancreatic cancer cell by upregulating c-Met phosphorylation and MAPK activity [49]. Irradiation of mouse embryo fibroblasts stimulates cancer cell repopulation in cell culture and xenograft models [50]. This effect is lost when the fibroblasts are deficient for caspase-3, a key executioner of programmed cell death. Moreover, radiotherapy is not unique in inducing pro-metastatic effects in preclinical models. Comparative analysis of matched colorectal cancer specimens shows that neoadjuvant chemotherapy results in increased presence of pro-invasive α-SMA-positive CAFs (cancer associated fibroblast) [51]. Monnier et al. demonstrated that after irradiation of the stromal bed, oral squamous cell carcinomas showed increased invasion and metastasis through the matricellular protein CYR61 [52]. Irradiation also has an effect on VEGF production and local angiogenesis, which finally contributes to metastasis formation [53].

Our results warrant further investigation of the potential pro-metastatic effects of radiation and indicate the need to develop efficient drugs which can be combined efficiently with RT in order to prevent therapy-induced spread of cancer cells. Nevertheless, the most efficient and simple solution is to prevent normal tissue irradiation. In breast cancer, the cardiopulmonary region can be spared of high doses by using multi-beam intensity-modulated radiotherapy or arc techniques, but often at the cost of a low dose spread [54]. Irradiating the breast when patients are in prone instead of supine position has been shown to spectacularly improve all lung dose-volume indices [55, 56].

## METHODS AND MATERIALS

### Cell lines

EA.hy926, a human endothelial cell line was obtained from ATCC (Manassas, VA, USA). MDA-MB-231GFP_luc, a human triple-negative breast cancer cell line [57]. 4T1_luc, a mouse triple-negative breast cancer cell was obtained from Sibtech (Brookfield, CT, USA). Beas-2B cell line, a human normal lung epithelial cell line, was kindly provided by Prof. K. De Bosscher (Cytokine Receptor Lab, VIB, Ghent University). EA.hy926, MDA-MB-231GFP_luc, and 4T1_luc cells were maintained in DMEM culture medium supplemented with 10% fetal bovine serum, 100 U/mL penicillin, 100 μg/mL streptomycin (Invitrogen, Waltham, MA, USA), and 2.5 μg/mL fungizone (Bristol-Myers, Squibb, Belgium). Beas-2B cells were maintained in MEM culture medium supplemented with 0,05% L-glutamine (w/v), 10% fetal bovine serum, 100 U/mL penicillin, 100 μg/mL streptomycin (Invitrogen), and 2.5 μg/mL fungizone. EA.hy926, 4T1_luc and Beas-2B were incubated with 5% CO_2_. MDA-MB-231GFP_luc cells were incubated with 10% CO_2_. Authenticity of ATCC cell lines was confirmed by short tandem repeat profiling in the last 6 months before use. Cell cultures were tested for mycoplasma contamination monthly by using MycoAlert Plus Kit (Lonza, Basel, Switzerland). MDA-MB-231GFP_luc, GFP expression was continuously induced with doxycycline (500 ng/mL, Sigma-Aldrich, St. Louis, MO, USA). 4T1_luc were selected with Zeocine (500 μg/mL, Invitrogen).

### Antibodies and reagents

Primary and secondary antibodies and reagents are described in Supplementary Materials and Methods.

### Conditioned medium of irradiated and unirradiated bronchial epithelial cells

Normal lung epithelial cells were cultured in a 25 cm^2^ culture flask. Cells were irradiated using a Small Animal Radiation Research Platform, SARRP system (X-ray tube:ISOVOLT 225M2 X-ray source; SARRP system, XStrahl®, Surrey, UK) at a constant rate of 3.45 Gy/min, for 174 seconds, thus receiving a single-fraction of 10.0 Gy (220 kV and 13.0 mA, using an 0.15 mm cupper filter and a 10 × 10 cm collimator). Cells were positioned at a source-to-surface distance (SSD) of 34 cm. Control “sham” samples (0 Gy) received similar handlings except for the irradiation. Conditioned medium containing soluble factors derived from irradiated epithelial cells (CM^LE_IR^) and medium of the sham epithelial cells (CM^LE^) was prepared as described in Supplementary Materials and Methods.

### Quantification DNA double-strand breaks (DSB)

Quantification of γH2AX is used to quantify DSB. For the *in vitro* experiments on human Beas-2B lung epithelial cells a mouse monoclonal anti-γH2AX primary antibody was used in combination with an Alexa488-conjugated rabbit anti-mouse secondary antibody and DAPI nuclear counterstain. The protocol as described in Depuydt et al. was followed [58]. For γH2AX foci analysis in the mouse lung tissue a rabbit polyclonal anti-γH2AX primary antibody was used in combination with a biotinylated goat anti-rabbit secondary antibody. To visualize the foci an immunoenzymatic staining using horse radish peroxidase-conjugated streptavidin and DAB was applied followed by haematoxylin counterstaining. The protocol as described in Bolcaen et al. was followed [59]. Mice were euthanized 15 minutes after receiving thoracic sham or 10 Gy irradiation.

### Viability assay

The viability was analysed with the LIVE/DEAD kit for mammalian cells (Invitrogen), as described in Supplementary Materials and Methods.

### Functional assays with direct cell-cell contacts

Cell growth assay. Beas-2B cells, cultured in DMEM with 10% FBS until 70% confluency, received a sham or 10 Gy radiation. After 24 hours 1 × 10^3^ MDA-MB-231GFP_luc or 4T1_luc cells were added to suspended and seeded together with 2 × 10^5^ sham or 10 Gy irradiated Beas-2B cells. After 4 days of co-culture, medium was changed and luciferine containing medium was added (150 μg/mL) 2 minutes before imaging. Imaging time was 2 min/cell plate. Light emitted from the breast cancer cells was detected by a highly sensitive CCD camera in the *In Vivo* Imaging System Lumina II (IVIS®, Caliper Life Science, Hopkinton, MA, USA). Analysis was achieved with Living Image® software (Caliper Life Science). There was a correlation between cell number and bioluminescence in vitro, using the *In Vivo* Imaging System (data not shown) [60].

Cell adhesion assay. To study the difference in adhesion of breast cancer cells to irradiated versus unirradiated epithelial cells, Beas-2B were cultured in a 6-well plate until confluency. The monolayer was sham or 10 Gy. 24 hours after treatment 2 × 10^4^ MDA-MB-231GFP_luc or 4T1_luc cells were added to the monolayers. To study the effect of AMD3100, a CXCR4 antagonist, cancer cells were pretreated for 30 minutes with 10 μM AMD3100. In AMD3100 condition, pretreated cancer cells were added to the monolayer with a total AMD3100 concentration of 10 μM. Adhesion was analyzed after 24 hours using IVIS as described above.

### Functional assays with conditioned media

Cell growth assay. MDA-MB-231GFP_luc and 4T1_luc cells (2 × 10^4^) were seeded in a 24-well plate and treated either with CM^LE_IR^ or CM^LE^. To study the effect of recombinant CXCL12 or MIF on cancer cell growth, cells were treated with CM^LE^ supplemented with MIF or CXCL12 (50 ng/mL). The effect of an inhibitor was studied by pretreatment of the cancer cells with 10 μM AMD3100 for 30 minutes and a total AMD3100 concentration of 10 μM in CM^LE_IR^. After 4 days cell numbers were analyzed by bioluminescent signal detection as described above.

Migration assay. MDA-MB-231GFP_luc cells (1 × 10^5^ cells) were plated in the upper compartment of a Transwell chamber (24-well insert, pore size 8 μm, Corning Incorporated, New York, NY), while in the lower compartment CM^LE_IR^, CM^LE^ or CM^LE^ supplemented with CXCL12 (50 ng/mL) was used as a chemoattractant. To study the effect of AMD3100, cancer cells were pretreated 30 minutes with 10 μM AMD3100, and AMD3100 was added in both compartments in CM^LE_IR^ with a final 10 μM concentration. Migration was stopped after 8 hours incubation and the insert was washed with PBS^D+^. Cells from the apical side were removed using a cotton swab before fixation with ice-cold methanol and DAPI staining. After 4 washing steps with PBS^D−^, the filter was mounted onto glass using glycergel mounting medium (Dako, Carpinteria, CA, USA). Cell nuclei were analyzed by counting 6 different, randomly chosen fields with a 10x objective on a Zeiss Axiovert 200M fluorescent microscope.

Transendothelial migration assay. Formation and analysis of endothelial monolayer is described in Supplementary Materials and Methods and Supplementary Figure S7.

Doxycycline-induced MDA-MB-231GFP_luc cells (1 × 10^5^) were added on top of the endothelial cells. CM^LE_IR^, CM^LE^ or CM^LE_IR^ + AMD3100 were used as a chemoattractant. After incubation for 24 hours the apical side of the chamber was washed twice with PBSD+ and scraped gently with a cotton swap. Migrated cancer cells, green fluorescent, were counted from 8 different, randomly chosen fields with a 10x objective on a Zeiss Axiovert 200M fluorescent microscope (Carl Zeiss, Micro-imaging, Heidelberg, Germany).

Morphology analysis. For quantification of morphological changes, 2 × 10^5^ MDA-MB-231GFP_luc single cells were seeded on glass cover slips in presence of control medium or CM^LE_IR^. After 4 days of incubation, cells were fixed with 3.7% formaldehyde for 20 minutes. Permeabilization with 0,1% Triton-X100 was done for 5 minutes and cells were blocked for 30 minutes, while shaking, with 1% BSA. Next, glasses were stained with F-actin stain phalloidin-Alexa Fluor 594 and DAPI and imaged with a Zeiss Axiovert 200M fluorescent microscope. Of each condition, 20 cells from 4 different glasses were used to score factor shape with the formula: (perimeter)^2^/(4 × π × area).

### Protein analysis

SDS-PAGE and Western blot analysis. Lysate preparation, SDS-PAGE and Western blot analysis are described in Supplementary Materials and Methods.

Cytokine array. RayBio®Label-Based human antibody array 507 (L-507, RayBiotech Inc., Norcross, GA, USA) was used to identify the cytokines playing a key role in the effect of irradiated bronchial epithelial cells. Cytokine array analysis is described in Supplementary Materials and Methods.

ELISA analysis. CXCL12 and MIF secretion levels were measured using quantitative immunometric sandwich enzyme immunoassays (ELISA = enzyme-linked immunosorbent assay), following the manufacturer's recommended procedures (R & D Systems, Minneapolis, MN, USA). Optical density was measured at 450 nm of wavelength, with correction set to 570 nm, on a PARADIGM™ Microplate Detection Platform (Beckman Coulter, Brea, CA, USA). Triplicate cultures of cells were tested for each experimental condition.

### Animal studies

Radiotherapy treatment planning. In this study, two different dose plans were set up and executed on the SARRP. One delivered 10Gy to the entire thorax, the other delivered 10 Gy to 10% volume of the right lung. First, dose distributions was calculated using the on-board CT as described earlier [61]. 4-week-old BALB/c female mice (Charles River, L'Abresle, France), were anesthetized, fixed on a plastic bed and placed on a holder secured onto the robotic positioning table. Cone-beam (CB) CT imaging is achieved by rotating the stage that supports the animal, horizontally between the stationary X-ray source and a flat-panel detector. The uncollimated primary beam, 20 cm × 20 cm at isocenter, is used for imaging. X-rays of 70 kV emitted from the 0.4 mm focal spot and filtered by 1 mm thick aluminum were employed. Images were acquired at a current of 1 mA, with “continuous” beam-on as well as “continuous” stage rotation. Three hundred and sixty (360) projections were acquired over 360°. Second, the treatment isocenter was placed in the lower part of the right hemisphere of the lung. For whole thorax treatment, two posterior-anterior beams, with a size of 9 × 3 mm and 10 × 10mm at isocenter, were used to cover the entire thorax (Supplementary Figure S5B). Treatment isocenter was set for both in the middle of both lungs. One posterior-anterior beam with a size of 3 × 3 mm was selected to irradiate 18 mm^3^ of the right lung with 10 Gy, which resembles approximately 10% volume of the right lung (8,57% calculation based on Knuts et al. [62]) (Supplementary Figure S5A, S5C). The CT scans are imported into Muriplan software (Xstrahl®). Next, image intensity-based tissue segmentation was performed to allow correct dose calculation throughout the different tissue densities. The voltage of the X-ray source is fixed at 220 kV with a tube current of 12 mA, emitted from the 3.0 mm focal spot and filtered by a copper filter of 0.15 mm. Mice with sham treatment only underwent the imaging part.

4T1_luc triple-negative mouse breast cancer model. All mice were orthotopically injected 24 hours after radiation treatment. One million 4T1_luc cells, suspended in a 100 μL mixture of serum-free DMEM and Matrigel (1:1), were injected into the mammary fat pad. The primary tumor volume was quantified as the product of caliper measurements of the longest and the shortest tumor diameter (V = 0,4 × (longest axis) × (shortest axis)^2^). Primary and metastatic tumor growths were monitored by bioluminescence. First, mice were giving an intraperitoneaal injection of 100 μL D-luciferin in PBS (150 μg/g mouse). Then, animals were anesthetized with 5% isoflurane in oxygen for induction and 1.5% isoflurane in oxygen for maintenance. Next, bioluminescent imaging was initiated 10 minutes after injection by a cooled CCD camera in the IVIS with a 15-cm field of view, binning factor of 8, 1/f stop and open filter. Exposure times were set automatically, depending on the luciferase signaling activity. ROIs were drawn for primary tumor and metastatic lesions and were calculated by the IVIS software, expressed in total flux (photon/s). Background photon flux was defined, for primary growth on a blanc mouse and for metastatic lesions on a normal lung, and extracted from all animal values. Images were initiated every 4 days after inoculation. After 4 weeks, mice were sacrificed and tumor and lungs were resected. Lungs were placed into 6-well plates and ex vivo bioluminescent imaging was performed by adding 300 μg/mL D-luciferin in excess. After imaging, all tissues were fixed in 4% buffered formalin. H&E staining and immunohistochemistry were performed using a NexES automated slide staining system (Ventana Medical Systems, Tucson, AZ) on paraffin sections. Animals were treated according to the European guidelines on animal experiments (2010/63/EU). They were kept in 12h light- dark cycles, with ad libitum access to food and water. Animal studies were approved by the Animal Ethics Committee of Ghent University, Belgium (ECD 10/36).

### Statistical analysis

Statistical analysis was performed using GraphPad Prism and confirmed by IBM SPSS Statistics 21.0 software. D'Agostino-Pearson was used for testing normal distribution. Normal distributed data were analyzed using unpaired *t*-test, adjusted with Welch's correction when variance was statistically different. All nonparametric data were analyzed using Mann-Whitney U test. All values in box- and whisker blots are expressed as the mean and 95% confidence interval. All other values are expresses as the mean ± SD. A *P* value of < 0.05 was considered statistically significant. Statistical tests were two -sided. All data are representative of at least three independent experiments.

## SUPPLEMENTARY MATERIAL FIGURES AND TABLE



## Abbreviations

α-SMA: Alfa smooth muscle actin
Akt: Protein kinase B
AMD3100: 1,1′-[1,4-Phenylenebis(methylene)]bis[1,4,8,11-tetraazacyclotetradecane]
BALB: Bagg Albino
BSA: Bovine serum albumin
CAF: Cancer-associated fibroblast
CCD: Charge coupled device
c-Met: Cellular mesenchymal to epithelial transition factor
CMLE: Conditioned medium lung epithelial cells
CMLE-IR: Conditioned medium irradiated lung epithelial cells
CT: Computed tomography
CXCL12: C-X-C chemokine ligand 12
CXCR4: C-X-C chemokine receptor 4
CYR61: Cysteine-rich angiogenic inducer 61
DAB: 3,3′-diaminobenzidine tetrahydrochloride hydrate
DAPI: 4′,6-diamidino-2-phenylindole
DiI: 1,1′-dioctadecyl-3,3,3′3′-tetramethylindocarbocyanine perchlorate
DMEM: Dulbecco's Modified Eagle's medium
DSB: Double strand break
ELISA: Enzyme-linked immunosorbent assay
ERK: Extracellular Signal-Regulated Kinase
GAPDH: Glyceraldehyde-3-phosphate dehydrogenase
G-CSF: Granulocyte colony-stimulating factor
GFP: Green fluorescent protein
Gy: Unit of absorbed dose and equivalent to 1 J/kg
H2AX: Histone 2A, member X
IVIS: In vivo imaging system
MAPK: Mitogen-activated protein kinase
MEK1/2: Mitogen-activated protein kinase/extracellular signal-regulated kinase kinase 1/2
MIF: Macrophage migration inhibitory factor
PBS D−: Phosphate-buffered saline without calcium and magnesium
PBS D+: Phosphate-buffered saline supplemented with calcium and magnesium
PRL: Partial right lung
ROI: Region of interest
RT: Radiation treatment/Radiotherapy
SARRP: Small animal radiation research platform
SD: Standard deviation
SDF-1: Stromal cell-derived factor 1
SPSS: Statistical package for the social science
STAT3: Signal transducer and activator of transcription 3
WT: Whole thorax
