# Supplementary material for: Radiation-induced lung damage promotes breast cancer lung-metastasis through CXCR4 signaling
Source: Oncotarget. 2015 Sep 15;6(29):26615–32. doi: 10.18632/oncotarget.5666 (PMC4694940; doi:10.18632/oncotarget.5666)
Supplement: Supplementary file 1 [file oncotarget-06-26615-s001.pdf]

Radiation-induced lung damage promotes breast cancer lung-metastasis through CXCR4 signaling

Supplementary Material

Supplementary Fig. S1

A

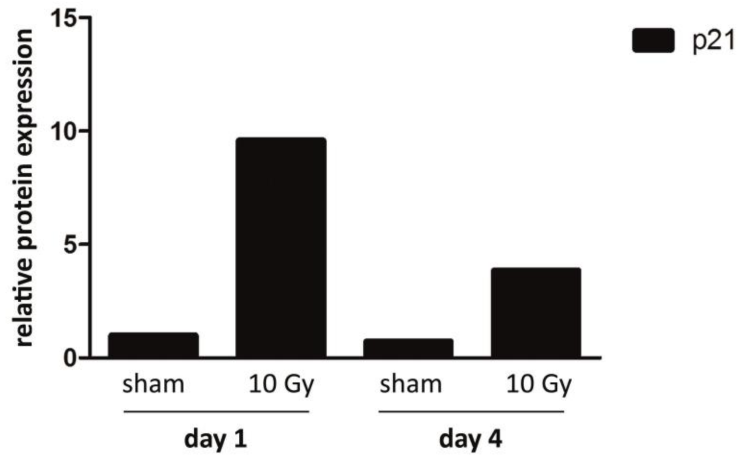

B

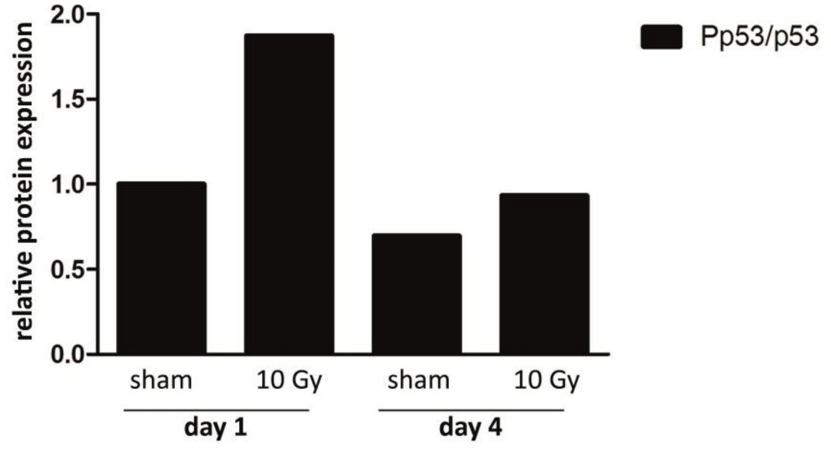

Supplementary Figure S1

Lung epithelial cells radiation response and senescence markers

A. Quantification of p21 protein expression level in irradiated and non-irradiated Beas-2B lysates made 1 and 4 days after treatment. Quantification normalized according the tubulin protein expression level. Protein levels relative to control, Beas-2B sham day 1.

B. Quantification of Pp53 and p53 protein expression level in irradiated and non-irradiated Beas-2B of lysates made 1 and 4 days after treatment. The ratio Pp53/p53 is shown in the graph. Quantification is normalized according the GAPDH protein expression level. Protein levels relative to control, Beas-2B sham day 1.

Supplementary Fig. S2

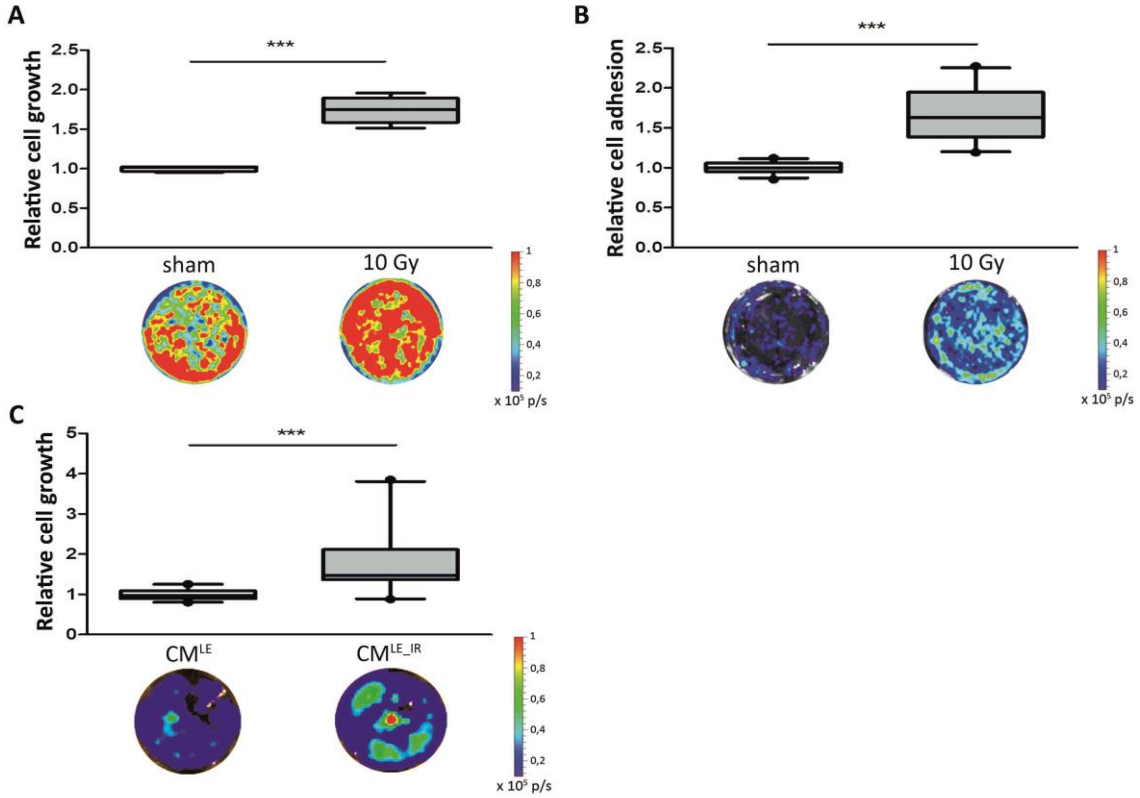

Supplementary Figure S2

Impact of irradiated lung epithelial cells on 4T1_luc breast cancer cell adhesion and growth

A. Box plots illustrating the relative cell growth of 4T1_luc cells . Co-culture of breast cancer cells with irradiated Beas-2B cells increases relative cell growth compared to co-culture with sham treated Beas-2B cells. Quantification by bioluminescent imaging after 4 days of incubation. Data is represented as relative fold change compared with the corresponding control value. 4T1_luc: *n*=6; ***, P<0.001 (Unpaired t-test with Welch's correction).
B. Box plots illustrating the relative cell adhesion of 4T1_luc cells. Relative adhesion of breast cancer cells to irradiated Beas-2B cell monolayer is increased compared to sham treated Beas-2B cell monolayer. Quantification by bioluminescent imaging after 24 hours incubation. Data is represented as relative fold change compared with the corresponding control value. 4T1_luc: *n*=10; ***, P<0.001. (Unpaired t-test with Welch's correction).

Impact of CM^{LE_IR} on breast cancer cell morphology, growth, migration and extravasation

C. Box plots illustrating the relative cell growth of 4T1_luc cells. Treatment of breast cancer with CM^{LE_IR} increases relative cell growth compared to cells treated with CM^{LE}. Quantification by bioluminescent imaging after 4 days incubation. Data is represented as relative fold change compared with the corresponding control value (CM^{LE}). 4T1_luc: *n*=18; ***, P<0.001 (Mann-Whitney U).

Supplementary Fig. S3

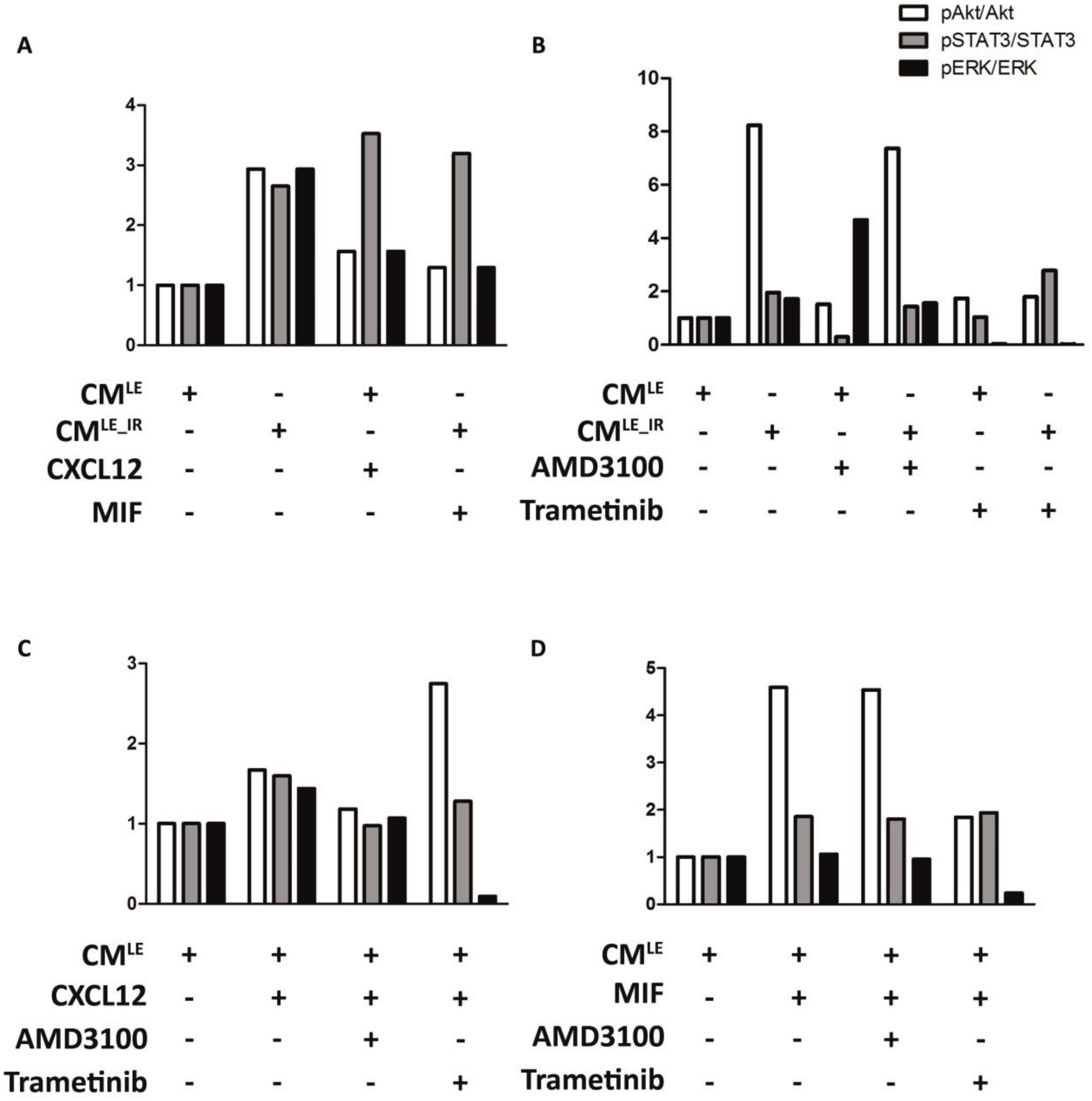

Supplementary Figure S4

Quantification pAkt/Akt, pSTAT3/STAT3 and pERK/ERK

Western blot analysis of the ratio pAkt/Akt, pSTAT3/STAT3 and pERK/ERK protein expression after tubulin normalization Protein levels relative to control MDA-MB-231GFP_luc cells exposed to CM^{LE} . For Western blot visualisation see Figure 6.

Supplementary Fig. S4

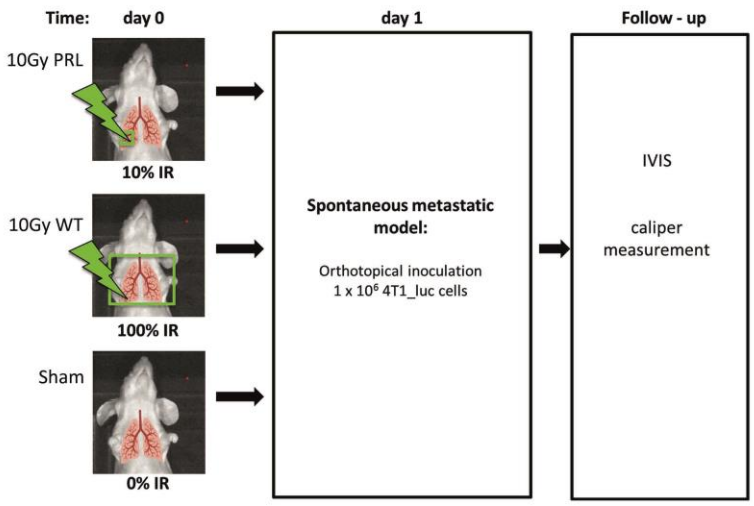

Supplementary Figure S4

In vivo experiment set-up

Supplementary Fig. S5

A

PRL

I. Set isocenter

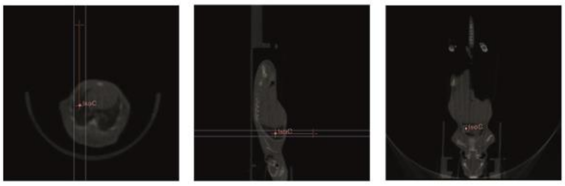

II. Segmentation

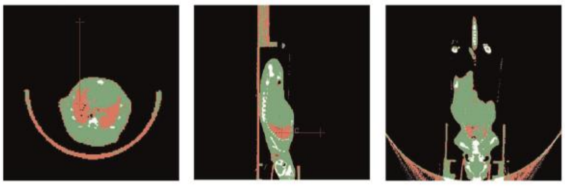

III. Dose distribution

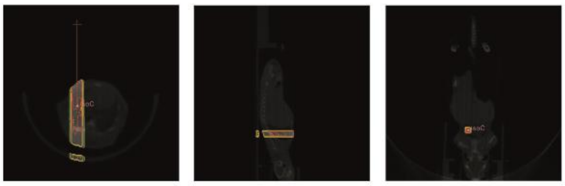

B

WT

I. Set isocenter

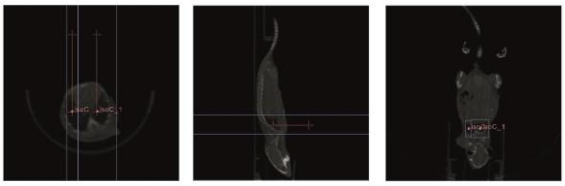

II. Segmentation

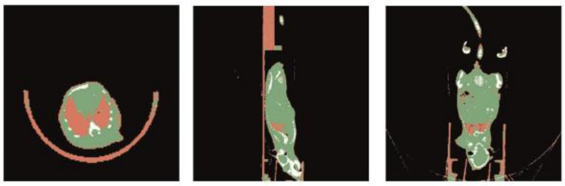

III. Dose distribution

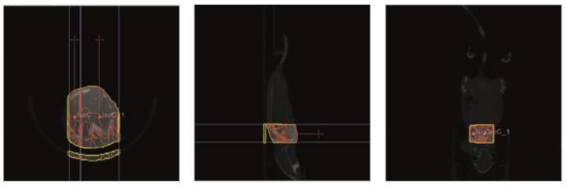

C

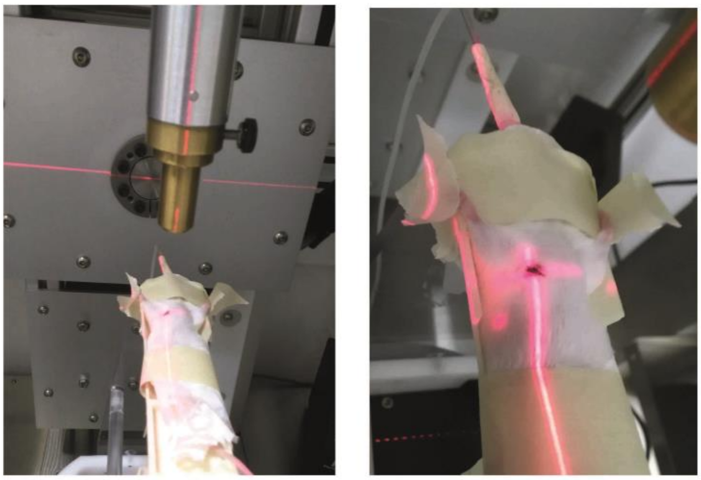

Supplementary Figure S5

SARRP protocol

A. SARRP protocol partial right lung irradiation. Accurate irradiation of the mouse was achieved by making an image of the lung by cone beam CT. Treatment isocenter was indicated on CT. Next, we mark the different kinds of tissue (segmentation) and the SARRP software calculates the mouse-specific dose.
B. SARRP protocol whole thorax irradiation. Accurate irradiation of the mouse was achieved by making an image of the lung by cone beam CT. Treatment isocenters were indicated on CT. Next, we mark the different kinds of tissue (segmentation) and the SARRP software calculates the mouse-specific dose.
C. Visualization isocenter partial lung irradiation. Mice were anesthetized, fixed on a plastic bed and placed on a holder secured onto the robotic positioning table. Laser beam indicates treatment isocenter.

Supplementary Fig. S6

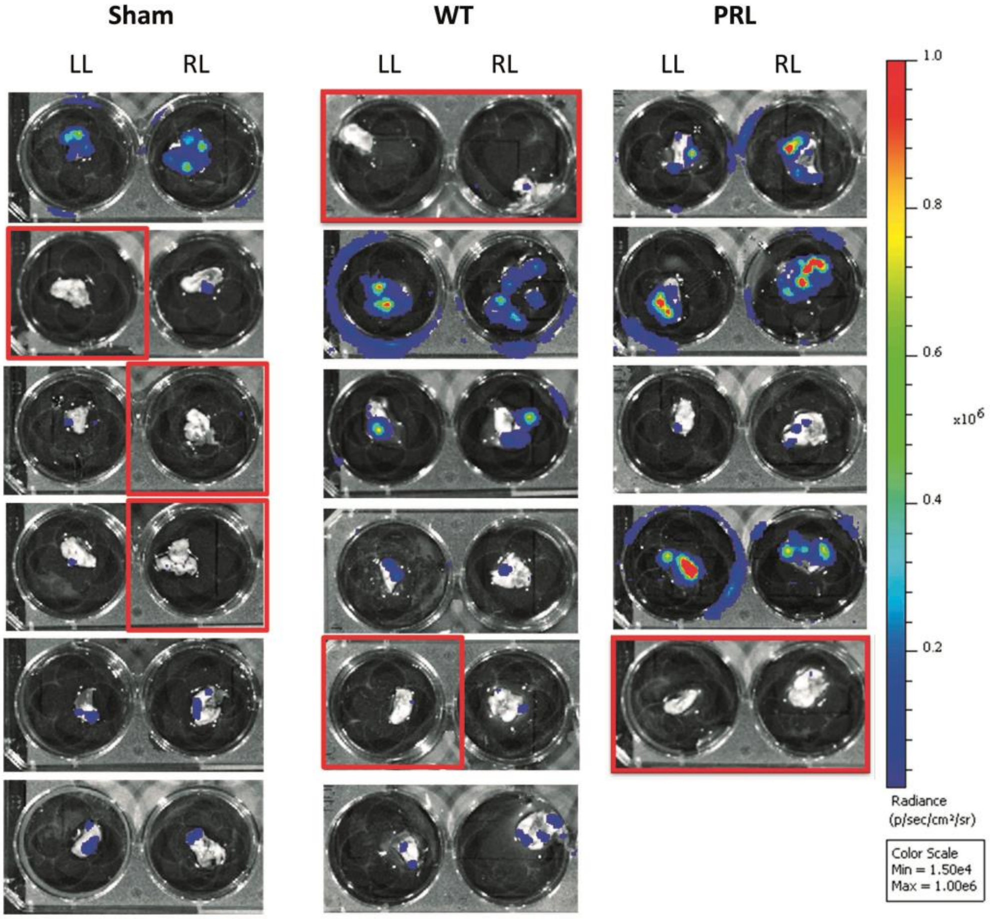

Supplementary Figure S6

Ex vivo set-up. Red square indicating no metastasis formation in the lung.

Supplementary Fig. S7

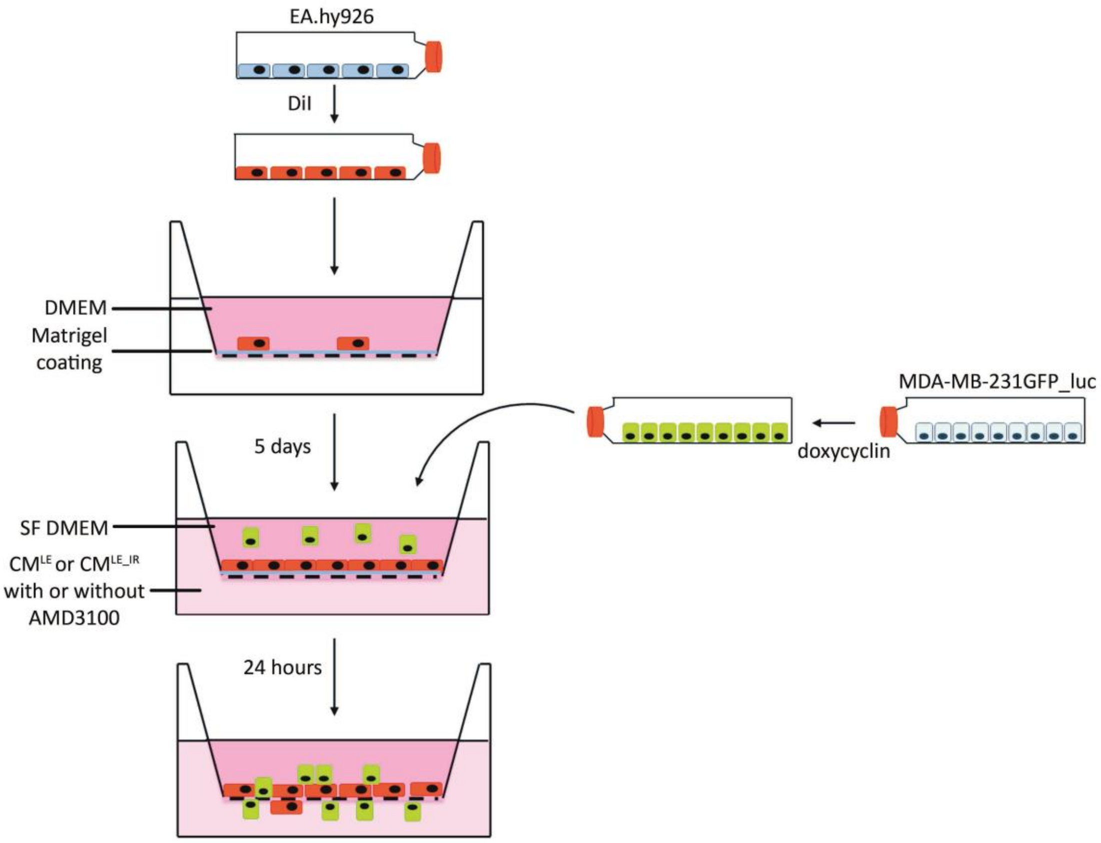

Supplementary Figure S7

In vitro functional assay: Transendothelial migration

Supplementary Materials and Methods

Antibodies and reagents

The following primary antibodies were used for Western blot analysis, IHC and ICC: rabbit polyclonal anti-Akt (9272, 1:1000), rabbit polyclonal anti-ERK1/2 (p44/42 MAPK, 9102, 1:1000), rabbit monoclonal anti-phospho-Akt (Ser473, 4058, 1:1000), rabbit polyclonal anti-phospho-ERk1/2 (p44/42 MAPK; Thr202/Tyr204, 9101, 1:1000), rabbit polyclonal anti-phospho-p53 (Ser15, 9284, 1:500), rabbit polyclonal anti-phospho-Stat3 (Tyr705, 9131, 1:1000), rabbit polyclonal anti-stat3 (9132; 1:1000) (Cell Signalling Technology, Danvers, MA, USA), rabbit polyclonal anti-CXCR4 (NB100-56437, 1:500) (Novus Biologicals, Cambridge, UK), rabbit polyclonal anti-p21 (H-164, sc-756, 1:500), mouse monoclonal anti-p53 (DO-1, sc-126, 1:1000) (Santa Cruz Biotechnology, Santa Cruz, CA, USA), mouse monoclonal anti-A-tubulin (T5168, 1:5000), mouse monoclonal anti-GAPDH (G8795, 1:5000) (Sigma-Aldrich, St. Louis, MO, USA) mouse monoclonal anti-ɣH2AX (613402, 1:500) (Biolegend, Sant Diego, CA, USA) rabbit polyclonal anti-ɣH2AX (00059, 1:3000) (Bethyl Laboratories, Montgomery, TX, USA) . Secondary antibodies coupled to horseradish peroxidase, Alexa-488 or Alexa-594 were obtained from Amersham Pharmacia Biotech (Diegem, Belgium) or Sigma-Aldrich. Nuclear staining DAPI stain, DAB (D5637) and F-actin stain phalloidin-Alexa Fluor 594 were from Sigma-Aldrich. Following reagents were used: AMD3100 octahydrochloride (3299) (Tocris Bioscience,Bristol, UK), Trametinib (S2673) (Selleckchem, Houston, TX, USA), recombinant MIF (289-MF-0002), recombinant CXCL12 (350-NS-010) (R&D Systems, Minneapolis, MN, USA), D-luciferin, Firefly, potassium salt (122796) (PerkinElmer, Waltham, MA, USA), Zeocine (E6110) (Promega, Madison, WI, USA) Doxycycline, FITC-Dextran (46945) (Sigma-Aldrich), Vybrant®DiI cell-labelling solution (V-22885) (Molecular Probes, Waltham, MMA, USA). In the *in vitro* experiments, AMD3100 was used at 10 µM, Trametinib at 50 nM,

recombinant CXCL12 and MIF at 50 ng/mL, D-luciferin at 150 µg/mL, Zeocine at 500 µg/mL, Doxycycline at 500 ng/mL, FITC-Dextran at 1 mg/mL and Vybrant®DiI 5µL/mL/10^6 cells. *In vivo* D-luciferin was used at 150 mg/kg mice and *ex vivo* at 300 µg/mL.

Viability Assay

Beas_2B cells were seeded in a 24-well plate. Plates were treated either with 10 Gy or 0 Gy. After 4 days cells were rinsed 3 times with PBS^{D+} and 200 µL of LIVE/DEAD mixture was added to the cells for 30 minutes at 37°C in 5% CO_2. Mixture contained 5 µM calcein-AM (living cells, green fluorescent 496/517nm) and 20 µM ethidium homodimer-1 (dead cells, red fluorescent 528/617 nm). Cells were washed with PBS^{D+} and analysed by fluorescence microscopy (Zeiss 510 META confocal laser scanning microscope, Carl Zeiss, Micro-imaging, Heidelberg, Germany).

Conditioned medium of irradiated and unirradiated bronchial epithelial cells

Three days after treatment, cells were washed, three times, and put on serum-free MEM culture medium with 100 U/mL penicillin, 100 µg/mL streptomycin, 2,5 µg/mL fungizone and put in an incubator at 37°C and 5% CO_2. After 24 hours the medium of both groups was harvested separately and centrifuged for 5 minutes at 1000 rpm on 4°C. The supernatants were collected and filtered through a 0.2 µM Whatmann filter. All conditioned media were normalized to an equal number of 6,25 x 10^5 cells. Both conditioned media were stored at -20°C until further use for experiments.

Protein analysis

Lysate preparation, SDS_PAGE and Western blot analysis

Lysates were made of 70% confluent cell cultures by PBS containing 1% Triton X-100, 1% NP-40 (Sigma), and the Halt® Protease and Phosphatase inhibitor cocktail (1:100, Thermo Scientific). After sonication and centrifugation of lysate (14000 rpm for 10 minutes at 4°C), supernatant was used for measuring protein concentration by using RC DC protein assay kit (Bio-Rad Laboratories S.A.-N.V.). Samples were prepared with an equal amount of protein in Laemmli sample buffer with 5% β-mercaptoethanol and 0.005% bromophenol blue. Next, samples were boiled for 5 minutes at 95°C. Finally, proteins were separated by electrophoresis. Proteins from the gel were blotted onto a nitrocellulose membrane and blocked in 5% nonfat milk in PBS^{D-} or 4% BSA in PBS^{D-} for phosphorylated proteins, both supplemented with 0,5% Tween-20 and immunostained. Next, a chemiluminescent substrate for horseradish peroxidase (ECL western blotting detection reagent; GE Healthcare, Belgium) was added and signal was measured and visualized by ProXima 2850 (Isogen Lifescience, De Meern, Netherlands). Quantification was done by ImageJ software.

Cytokine array.

Assay for cytokine antibody arrays was performed as per manufacturer's instructions. Briefly, cytokine array membranes were blocked with 5% BSA/TBS (0.01 M Tris HCl pH 7.6/0.15 M NaCl) for 1 hour. Membranes were then incubated with about 2 ml of CM^{LE} or CM^{LE_IR} after normalization with equal amounts of protein. After washing 3 times with PBS^{D-} supplemented with 0.1% Tween 20, the membranes were then incubated with a cocktail of biotin-labelled antibodies against different individual cytokines. The membranes were then washed and incubated with HRP-conjugated streptavidin (2,5 pg/ml) for 1 hour at room temperature. After 2 times wishing with PBS^{D-} supplemented with 0.1% Tween 20, the

signals were detected by ProXima 2850. Densitometric values of spots were quantified using ImageJ Software (Scion Corp., Frederick,MD, USA).

Functional assays with conditioned media

Transendothelial migration assay

Endothelial monolayer formation and quantification. EA.hy926 endothelial cells were transiently red-fluorescently labeled with Vybrant®DiI cell-labelling solution according to the manufacturer's protocol. Endothelial cells were seeded in the upper compartment of a Matrigel (100 µg/mL) coated 8 µm pore size Transwell culture system and cultured in normal cell conditions at 37°C in 5% CO_2 for 5 days until a monolayer was formed. Monolayer permeability was tested by fluorescent measuring of 70 kDa FITC-dextran flux across the endothelial cell monolayer. Briefly, the endothelial monolayer was washed 2 times with PBS^{D+} followed by addition of 150 µL of FITC-Dextran (1 mg/mL) to the upper chamber and 700 µL PBS^{D+} in the lower chamber. After 1 hour incubation at 37°C in 5% $CO_{2,}$ FITC-Dextran concentration from each lower chamber was calculated using a fluorescent multi-well plate reader with excitation and emission wavelength of 485 nm and 530 nm, respectively (SpectraMax® Paradigm® Multi-Mode Microplate Detection Platform reader, Molecular Devices, Sunnyvale, California, USA). When no fluorescent intensity was detected, EA.hy926 reached confluency.

Supplementary Table 1

		CM^{LE}				CM^{LE_IR}			
array number	cytokine	cytokine expression		mean expression	relative expression	cytokine expression		mean expression	relative expression
470	TGF-beta 5	8513.368	8832.832	8673.1	1	8202.468	8244.489	8223.4785	0.948159078
40	BIK	6092.974	5131.104	5612.039	1	4995.983	6219.761	5607.872	0.999257489
249	IL_1 sRII	7862.589	7671.711	7767.15	1	7864.276	7685.953	7775.1145	1.001025408
438	S100A10	7717.418	8304.125	8010.7715	1	8503.296	7887.347	8195.3215	1.023037731
216	ICAM-2	6980.681	7628.196	7304.4385	1	6694.811	8961.761	7828.286	1.071716327
336	LECT2	3083.841	3723.196	3403.5185	1	3874.054	3677.983	3776.0185	1.109445563
78	Csk	4489.619	4410.933	4450.276	1	6515.74	6732.983	6624.3615	1.48852824
206	HCR / CRAM-A/B	873.477	789.184	831.3305	1	1598.912	1601.175	1600.0435	1.924677971
120	Endostatin	3074.69	3347.004	3210.847	1	7548.61	6923.945	7236.2775	2.253697389
430	P-selectin	7919.246	7966.61	7942.928	1	20156.73	20934.73	20545.731	2.586669676
199	GREMLIN	566.113	586.749	576.431	1	2081.841	1301.276	1691.5585	2.934537698
335	LBP	2341.255	2459.861	2400.558	1	9519.64	9708.711	9614.1755	4.004975302
357	M-CSF	546.87	677.456	612.163	1	3443.012	4220.205	3831.6085	6.259131146
370	MMP-1	1301.527	271.728	786.6275	1	6794.054	4673.397	5733.7255	7.288997016
194	Glypican 3	1001.234	1021.234	1011.234	1	6704.882	10301.37	8503.125	8.40866209
200	GRO	284.678	120.142	202.41	1	2064.477	1606.551	1835.514	9.068297021
367	MIP 2	374.263	252.556	313.4095	1	2876.184	3550.184	3213.184	10.25235036
488	TIMP-3	468.678	350.678	409.678	1	4119.154	5167.497	4643.3255	11.33408555
353	MCP-1	201.849	303.142	252.4955	1	2530.113	3555.962	3043.0375	12.05184845
471	TGF-beta RI/ ALK-5	624.163	988.77	806.4665	1	9708.953	9837.146	9773.0495	12.11835768
346	LRP-6	390.142	384.406	387.274	1	5943.803	4609.054	5276.4285	13.62453586
229	IGFBP-rp1/IGFBP-7	1221.426	1383.841	1302.6335	1	20889.75	18105.68	19497.7165	14.96792191
373	MMP-7	266.385	120.192	193.2885	1	2923.012	2974.74	2948.876	15.25634479
527	VEGF	194.556	152.071	173.3135	1	2569.326	2745.962	2657.644	15.33431614
487	TIMP-2	660.941	350.213	505.577	1	7919.468	7820.175	7869.8215	15.56601962
186	GRF alpha-4	358.678	349.263	353.9705	1	5975.811	5543.083	5759.447	16.27098021
176	GDF3	109.263	108.021	108.642	1	1799.69	2279.154	2039.422	18.77194823
260	IL-6	286.556	238.728	262.642	1	4280.581	5634.569	4957.575	18.8757891
233	IGF-II R	279.435	169.021	224.228	1	4491.811	4607.882	4549.8465	20.29116123
60	CCR3	430.627	193.314	311.9705	1	6979.782	5819.447	6399.6145	20.51352452
188	GITR Ligand / TNFSF18	354.385	275.728	315.0565	1	5459.447	9262.418	7360.9325	23.36384902
15	ALCAM	283.971	264.799	274.385	1	7537.983	5343.083	6440.533	23.4726133
477	Thrombospondin-1	699.891	726.77	713.3305	1	16009.97	19078.93	17544.4535	24.59512596
181	GDF-15	41.778	86.192	63.985	1	1711.74	1700.527	1706.1335	26.66458545
262	IL-7	121.314	137.778	129.546	1	4759.276	3448.861	4104.0685	31.68039538
278	IL-13	216.435	208.263	212.349	1	6133.882	7389.518	6761.7	31.84239153
277	IL-12R beta 2	192.728	67.657	130.1925	1	4863.376	5012.083	4937.7295	37.92637441
208	GLO-1	96.971	163.607	130.289	1	5684.468	5095.045	5389.7565	41.3677018
133	FAM38	204.142	171.335	187.7385	1	8978.468	6982.033	7980.2505	42.50726676
522	uPA	107.607	277.092	192.3495	1	9242.296	8372.882	8807.589	45.78950816
345	LRP-1	161.728	77.314	119.521	1	6656.368	4831.276	5743.822	48.0570109
151	FGF-16	81.071	70.607	75.839	1	3615.619	3987.054	3801.3365	50.12376877
445	sFRP-4	226.778	264.506	245.642	1	12691	12164.76	12427.8825	50.59347546
372	MMP-3	167.142	70.071	118.6065	1	6900.539	5825.761	6363.15	53.64925194
292	IL-17RC	71.899	117.192	94.5455	1	5902.497	5907.104	5904.8005	62.45459065
104	Dkk-1	167.263	71.778	119.5205	1	6770.761	8387.903	7579.332	63.41449375
225	IGFBP-2	125.485	98.485	111.985	1	6617.882	7606.861	7112.3715	63.51182301
486	TIMP-1	117.607	147.021	132.314	1	8509.459	8343.782	8426.6205	63.68653733
499	TNF RI/ TNFRSF1A	96.021	76.485	86.253	1	5970.004	5723.468	5846.736	67.78588571
384	MMP-20	123.192	34.485	78.8385	1	5821.175	7018.782	6419.9785	81.43202243
**442**	**SDF-1/CXCL12**	**100.485**	**70.607**	**85.546**	**1**	**7977.418**	**6373.539**	**7175.4785**	**83.87859748**
110	EDA-A2	50.071	83.778	66.9245	1	5664.326	5576.146	5620.236	83.97875218
**362**	**MIF**	**145.607**	**84.192**	**114.8995**	**1**	**10053.27**	**9814.853**	**9934.06**	**86.45868781**
293	IL-17RD	81.314	82.778	82.046	1	9926.782	7834.418	8880.6	108.2392804
282	IL-15R alpha	74.899	82.778	78.8385	1	9325.246	7908.66	8616.953	109.2987944
264	IL-8	57.364	58.192	57.778	1	7876.447	7077.589	7477.018	129.4094292
173	GCSF	75.192	66.485	70.8385	1	9783.095	10946.51	10364.8025	146.3159511
228	IGFBP-6	39.364	70.364	54.864	1	9958.062	9769.184	9863.623	179.7831547
169	Galectin-3	33.364	68.071	50.7175	1	9885.095	11372.56	10628.8275	209.5692315

	equal expression
	1-25 fold overexpression
	26-50 fold overexpression
	> 50 fold overexpression

Table 1 Cytokine expression levels. ImageJ quantification of cytokine array. CM^{LE} values were used as control and compared with CM^{LE_IR}.
